# Visuospatial, oculomotor, and executive reading skills evolve in elementary school, and errors are significant: a topological RAN study

**DOI:** 10.3389/fpsyg.2024.1383969

**Published:** 2024-06-06

**Authors:** Mario Lecce, Daniela Miazza, Carlo Muzio, Maria Parigi, Alessandra Miazza, Mattia G. Bergomi

**Affiliations:** ^1^Gruppo AppRendiMente, Pavia, Italy; ^2^Independent Researcher, Milan, Italy

**Keywords:** visuospatial skills, rapid automatized naming, crowding, antigrouping, monitoring, executive functions, where-centric, where-attention

## Abstract

We investigate the development of visuospatial and oculomotor reading skills in a cohort of elementary school children. Employing a longitudinal methodology, the study applies the Topological serial digit Rapid Automated Naming (Top-RAN) battery, which evaluates visuospatial reading skills leveraging metrics addressing crowding, distractors, and voluntary attention orientation. The participant pool comprises 142 students (66 males, 76 females), including 46 non-native speakers (21 males, 25 females), representing a diverse range of ethnic backgrounds. The Top-RAN dataset encompasses performance, error, and self-correction metrics for each subtest and student, underscoring the significance of these factors in the process of reading acquisition. Analytical methods include dimensionality reduction, clustering, and classification algorithms, consolidated into a Python package to facilitate reproducible results. Our results indicate that visuospatial reading abilities vary according to the task and demonstrate a marked evolution over time, as seen in the progressive decrease in execution times, errors, and self-corrections. This pattern supports the hypothesis that the growth of oculomotor, attentional, and executive skills is primarily fostered by educational experiences and maturation. This investigation provides valuable insights into the dynamic nature of these skills during pivotal educational stages.

## 1 Introduction

### 1.1 Problem

Throughout their academic journey, students are expected to be able to quickly and easily shift their focus to the relevant course content, whether it’s on a whiteboard or in a book. However, it’s important to note that individuals vary in their ability to accurately and efficiently locate people, objects, and words. Struggles with reading, writing, or copying from a book or blackboard can be attributed to several factors, including inadequate teaching, cognitive difficulties, visual problems, or attention deficits. Research has shown consistent, independent variations in spatial gaze localization skills among individuals beyond those influenced by age, sex, and other external factors. While average adult readers exhibit domain-specific oculomotor reading performance, individual differences in specific reading domains evolve dynamically in children between six and eleven years old. Studies have also shown that general saccadic abilities, particularly voluntarily controlled saccades, develop and reliably differentiate the performance of people with dyslexia from that of average readers during development. The evolution of voluntary components of oculomotor computation can capture differences between individuals, but are these voluntary components of the saccadic computation used while reading? According to Feng (2012), voluntary saccadic programming is solely related to cognitive-linguistic control. The basis for oculomotor computation in reading lies in the visuospatial qualities of horizontally aligned words. While in a passive task, the orientation of attention and gaze can be strongly determined by the visuospatial attributes of the environment, in the active reading task, the subject must move their gaze from left to right until the end of the line and then move on to the following line, gathering valuable information for language processing on each fixation. In light of the above, the question arises as to whether language processing skills solely influence the evolution of voluntary eye movements during reading or if there are other cognitive abilities, such as voluntary oculomotor computations and visuospatial index/tags arrangement, that develop and can be improved through learning. Do saccadic spatial skills and overt visual attention shifting along the text progressively improve throughout schooling, or are they simply a result of improved linguistic processing of the text but remain unchanged even if individually differentiated? Assuming that the saccadic spatial abilities hypothesized above exist from first grade through the end of elementary school, we should measure evolution in the ability of eye movements in reading to quickly and accurately locate target words/digits to be decoded, even in the presence of localization difficulties brought about by distractors or crowding.

### 1.2 Aim

In pursuit of uncovering the evolution of oculomotor mechanisms underlying word location in schoolchildren, our investigation delves into the ability (and development) of these young minds to orient their gaze horizontally and accurately locate words within a text, regardless of the arrangement of words on the page (beginning, middle, or end of a line). By doing so, we aim to determine if subjects can successfully orient their gaze laterally without incurring in excessive vertical errors when identifying words. We are also interested in determining whether children can precisely scan texts with multiple lines correctly, go to the following line, and perform precise voluntary regressions to re-read a word by the end of first grade. Another question is whether individuals acquire these skills through implicit learning, thus improving their speed and accuracy by simply performing reading tasks.

Answering these questions, we suggest using an experimental paradigm to measure spatial localization skills in a highly ecological manner. The experimental paradigm should be close enough to the everyday reading experience to enable the reutilization of the voluntary orienting of attention required by reading. At the same time, the paradigm should expose the voluntary oculomotor-visuospatial orientation of attention (OVO) to visuospatial difficulties to test its accuracy, speed, error monitoring, and, ultimately, the degree of automation.

### 1.3 Contribution

With these goals in mind, we first reviewed the literature concerning the development of oculomotor computation and spatial localization in the reading environment in elementary grades through adulthood and the literature on Rapid Automated Naming (RAN) concerning the same topics.

Secondly, we designed a battery of Serial Rapid Automated Naming (RAN) tasks called Topological RAN (Top-RAN) with three purposes: (1) Maintain a reasonable degree of ecological proximity to the reading task; (2) Make lexical access extremely easy through the use of digits, which are the most rapidly automated characters in the course of schooling; (3) Parameterize and modulate the difficulty introduced by the digits’ spatial localization. Modular digit arrangements allow us to maintain the task ecological enough to resemble the typical RAN. To this aim, we used crowding, distractors, and differential perceptual salience to make spatial localization difficult.

The proposed protocols allow us to shed some light on the mechanics underlying the development of oculomotor, attentional, and executive reading skills. We devised an administration protocol and acquired data from primary schools. Consequently, we present a new dataset describing performance, error, and self-corrections per subtest and student throughout the acquisition process. Furthermore, we devised and implemented a Python package to guarantee the reproducibility of the results presented in the remainder of this manuscript. In particular, the analysis pipelines allow the cluster detection of the student cohort by performance and error on a subtest basis, detect possible biases affecting the subtests, and provide a similarity measure between subtests.

Administration of the Top-RAN battery could reveal only one of the following scenarios: (A) Visuospatial-oculomotor difficulties neither exist nor evolve. (B) Visuospatial-oculomotor difficulties differ according to the task but lack evolution/learning. (C) Visuospatial difficulties differ according to the task and evolve, producing not only a progressive and differentiated reduction in execution times but also an equally progressive and differentiated reduction in errors and self-corrections.

### 1.4 Review structure

In the literature review, we will begin by emphasizing the evolution of oculomotor behavior expressing spatial localization skills in reading, its origins, and its domain specificity. We will then give a general overview of perceptual span in reading, its evolution, and its visuospatial components, especially regarding word length span. In this part of the discussion, we will highlight the special significance of the extreme right side of the perceptual span. In the section Word Targeting and Spatial Indexing in Reading, we will discuss what is known about the mechanisms of word targeting, carriage return saccades, regressions, and the use of spatial indexes in regressions and oculomotor return inhibition. We will then move on to discuss the precursors of our experimental paradigm. We will begin by discussing the automated rapid naming and the visual scanning hypothesis. Then, we will discuss errors and self-corrections for performance evaluation in all RAN tasks and similar oculomotor paradigms. In the subsection The DEM Test, we will expound on the history and rationale for this paradigm, the criticisms it has been subjected to, and its limitations. Finally, we will expose our viewpoint and the basic choices implemented in the experimental paradigms belonging to the Top-RAN battery.

## 2 Review

### 2.1 Maturation of the saccadic network, interaction with higher cognitive control, and domain specificity of reading eye movements

Saccades are rapid, ballistic eye movements (not modifiable after their instantiation), which serve to place the fovea (i.e., the area of maximum visual acuity of the retina) on objects of interest for their high-resolution perceptual processing. Each *data collection pause* is called a *fixation*. Each fixation is valuable for computing the next saccadic target. In this way, perception, attention, and oculomotion need continuous coordination, especially in tasks that require voluntary control, such as reading.

Eye-tracking techniques have made it possible to assess the evolution of reading eye movements in children. Several studies based on eye tracking have found a progressive reduction in sentence reading times, fixation duration, number of fixations and regressions, and the probability of regression itself, increasing saccadic amplitude and word skipping probability (e.g., Rayner, 1986; Häikiö et al., 2009; Huestegge et al., 2009; Blythe et al., 2011). However, children of different ages present relevant differences in their oculomotor behaviors.

Frequently, the evolution of eye movements during reading in children is attributed to two macro-factors: on the one hand, the maturational components of the neurological network controlling saccades. On the other hand, the interaction of the saccade neurological network with higher cognitive control, especially language processing. The two factors are considered in a relationship of complete subordination of the former to the latter. The weight of cognitive control on the evolution of oculomotor reading behavior has been confirmed through software simulations of eye movements in adults and children obtained by applying reading eye movement models such as the E-Z Reader (Reichle et al., 2013). The simulation of children’s reading eye movements was very similar to reality when the overall lexical processing time was prolonged in the model but not when the accuracy and timing parameters of oculomotor computations were altered.

Neurological maturation of the saccadic network is likely to contribute to improving reading skills. Some parameters of saccadic eye movements performed in tasks other than reading, such as peak saccadic velocity (e.g., Salman et al., 2006), saccadic gain (Cohen and Ross, 1978) and saccadic amplitudes (Huestegge et al., 2009) have a rapid evolution because they are under the control of centers mainly located in the brainstem and cerebellum that develop early (e.g., Salman et al., 2006). According to Kooiker et al. (2016), saccadic eye movements visually guided and elicited by salience have rapid development (for targets with maximum salience, it is completed at around age four; for those with low salience, around nine; see also Blakley et al., 2022). However, we know that frontal and parietal cortical areas are involved in voluntary gaze orientation tasks (such as reading). These areas take longer to mature (for a review of the literature on the development of eye movement control, see Luna et al., 2008). Nevertheless, how does the maturation of these cortical areas evolve when it comes to reading? Does the maturation of the cortical saccadic network involved in reading follow the lines of evolution shared by all saccadic tasks, or are there domain-specific specializations? In this regard, the pioneering work of Goold et al. (2019) on studying the oculomotor cortical network involved in reading is illuminating. The authors acquired fMRI and eye-tracking readings of subjects performing three types of oculomotor tasks: one of natural reading of a text and two of simulated reading (“move your eyes as if you were reading”) of pseudo-words and consonant strings. Thanks to studies that identified the cortical network of eye movement control using single saccade paradigms (e.g., Everling and Fischer, 1998; Munoz and Everling, 2004; Jamadar et al., 2013), the authors were able to choose specific areas whose activations they compared. They found that bilateral lFEFs and bilateral SEFs reliably differentiated between reading and pseudo-reading tasks. We know that FEFs play a cognitive control role in the antisaccade task (e.g., Jamadar et al., 2013), and SEFs are involved in executive functions (e, g., Stuphorn and Schall, 2006). Goold et al. (2019) argue that these areas of the oculomotor reading network derive specificity from interaction with cognitive systems. In other words, they argue for the domain-specificity of reading eye movements because beyond their behavioral similarity to other tasks, reading saccades subtend specific oculomotor networks that are different from those activated by other, even very similar, tasks.

How does the cognitive-linguistic network relate to the oculomotor network of reading? Does it only control certain parts of the oculomotor network, or does it also involve learning other oculomotor-attentive skills for better reading?

### 2.2 Saccadic computation and spatial qualities of text: word length span

The perceptual span is the number of characters in a line of text to the left and right of a fixed letter, from which information necessary for fluent and effective text reading is acquired. Fluent and effective reading is free of slowdowns or other strategic adaptations of oculomotor reading performance to the experimental paradigm. In order to analyze the span, two gaze-contingent experimental paradigms were mainly used: the moving window (McConkie and Rayner, 1975; Rayner, 2014) and the boundary paradigm (Rayner, 1975). Researchers have defined many span characteristics through their application in average adult readers.

Span, in a reading task, extends only over the currently read row and not over adjacent rows (although the experiments performed did not test for the influence of alterations in the upper rows, but only in the lower ones; e.g., Pollatsek et al., 1993). This information is crucial because it also delimits the text space from which the information for calculating upcoming saccades is extracted.

In early publications in the 1970s and 1980s, it was found that in alphabetic scripts that read from left to right, the span is 3–4 characters to the left of fixed letter and extends to about 14–15 letter spaces to the right of fixation (Rayner, 1998). This asymmetry has also been demonstrated in children (Sperlich et al., 2015) and has been shown to reverse for reading languages that proceed from right to left (e.g., Hebrew script, Pollatsek et al., 1981; Arabic script, Zhou et al., 2021). It should be noted that later studies have determined that the perceptual span of a particularly efficient adult can exceed 15 characters to the right of fixation (e.g., Häikiö et al., 2009; Choi et al., 2015) to the entire line (Veldre and Andrews, 2014).

There are relatively few studies on the development of perceptual span from early schooling to adulthood. Rayner’s (1986) pioneering study, using the moving window paradigm and symmetrical windows, finds that in the group of seven- to nine-year-old children (12 subjects), smaller windows of 11 + 11 characters around fixation alter performance, while from age 12 to adulthood (12 subjects) only a window of 14 + 14 characters around fixation has no impact on reading performance: thus impairment of all information available outside the window (letter identity and characteristics, spatial location of words) from ages seven to nine is irrelevant only for huge windows (11 + 11 characters). The development of this complete information window from age 12 onward is minimal (only three characters per side). Furthermore, by altering the content of words following the fixation, Rayner finds that the span of words to the right of the fixation for 7-year-olds is one word, while for 9- and 11-year-olds and adults, the span is 2. Rayner concludes that 7-year-old readers pre-process word length information with greater eccentricity around fixation than pre-process letter-specific information. The results of Häikiö et al. (2009) were conducted with the moving window paradigm on a sample of 80 Finnish subjects. They detect a progressive expansion of the letter identity span (i.e., the number of fully identified letters). At age 7, children identify five letters to the right of fixation; at age 9, seven letters; and from age 11 to adulthood, nine letters. The authors, also find that the span of the letter feature (number of characters to the right of fixation of which information can be gathered on at least the coarse features: ascending, descending, shape of the letter body) at age 11 and in adulthood is greater than the span of letter identity, being 11–12 characters. Unfortunately, the study by Häikiö et al. (2009) and subsequent studies on the evolution of perceptual span by Sperlich et al. (2015, 2016) did not measure word length span because the experimental paradigm used does not elide spaces between words outside the moving window, as Rayner (1986) had done.

**Figure 1:**
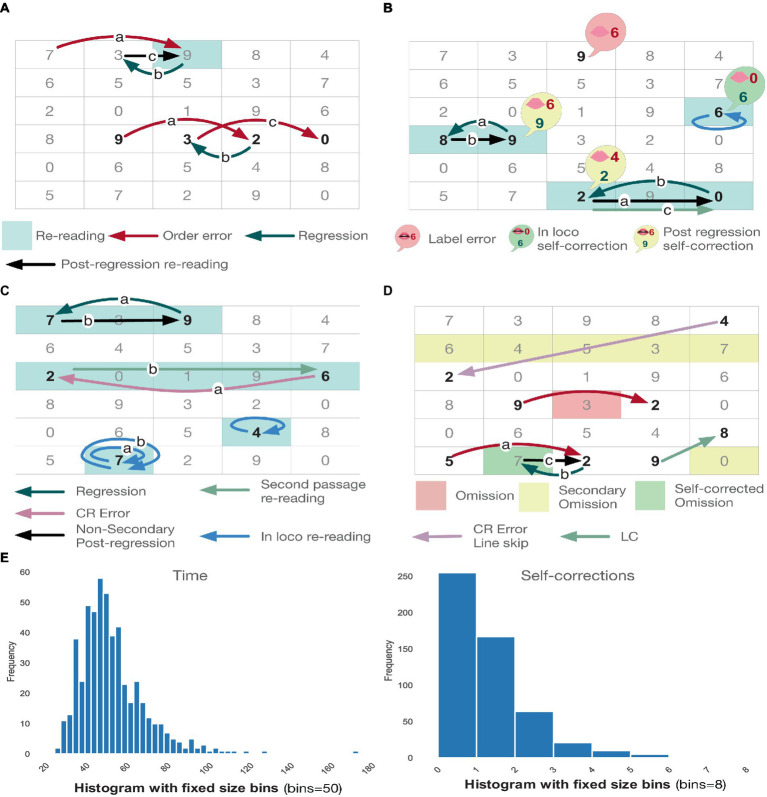
**(A)** Shows examples of order error (first case [7-9-3] with autocorrection). **(B)** Shows examples of label pronunciation error (first case not self-corrected). **(C)** Shows examples of re-readings. **(D)** Shows examples of omissions. **(E)** Showcases the execution time and self-correction histograms for the entire dataset.

The word-length span, which has its basis in the seminal work of Rayner (1975), consists mainly of the low-frequency spatial information collected during the single fixation and from the parafoveal retinal area. This information is available after 50 ms of eye-to-brain lag from the onset of fixation (see E-Z Reader model, Reichle, 2011; Reichle and Sheridan, 2014) and relates to word boundaries and word length (in addition to word shape and initial letters; Rayner et al., 2004). Research with moving windows and removal of inter-word spaces or their replacement with fillers has shown that both word identification and eye-movement control are hindered, producing significant impairments in fluency (e.g., Rayner et al., 1998). In Rayner’s (1986) study, the evolution of word length span, which does not stop in sixth grade, is evident in Figure 1 on page 219 and Table 3 on page 220 of his article.

Research still needs to fully clarify what functions are hypothesized for the extreme right part of the perceptual span (in Western scripts that read from left to right) as measured by the moving window technique in the average adult reader. Indeed, we consider the following: (1) maximum 9 characters to the right of fixation are acquired parafoveally for complete letter identification (Rayner, 1975; Häikiö et al., 2009), (2) maximum 11–12 characters to the right of the fixation convey information about coarse features of letters (Häikiö et al., 2009), (3) adult perceptual span measured by moving window can exceed 15 characters, going as high as 23 characters or the whole line to have no alteration in reading compared to reading without window (e.g., Choi et al., 2015), (4) spatial information regarding saccadic computation for impending target words does not fully justify this span size because in the average adult reader the saccades have a preferred length of 7 ± 2 characters (Reichle et al., 2003); (5) the fact that foveal loading (higher or lower frequency of the fixed word) is able to dynamically modulate perceptual span as early as second grade, as shown by Meixner et al. (2022), does not exclude that span in the face of high foveal loading uniformly reduces all its components: both acquisition span (span of letter identity + span of letter feature) and localization span (span of word length).

Thus, from a non-negligible part of the adult’s perceptual span (3 to 11--or more--character spaces) would come information essential for maintaining maximum reading performance, and this information is in no way used to identify letters or words, but only to localize them. Therefore, we hypothesize that a part of attentional deployment is devoted to confirmatory localization of the future path and its collinearity. The words contained in the far right part of the perceptual span could perform their stabilizing function as landmark objects necessary for the confirmation of the correct spatial localization of the target of the next saccade after its landing with the purpose of perceptual continuity and facilitation of post-saccadic error monitoring as conceptualized in the Deubel (2004) and Deubel et al. (1998) studies.

### 2.3 Word targeting and spatial indexing in reading

A detailed examination of the literature pertaining to saccadic word targeting, line-initial return saccades, short- and long-range regressions, and Oculomotor Inhibition of Return is deferred to Supplementary materials. This part of the literature adds little to our knowledge about the proactive/predictive part of the span (to the right of the fixed word in Western alphabetic scripts) in terms of accuracy and evolution. So we ask: Is the ability to deploy proactive/predictive spatial indices readily available with maximum efficiency from the earliest elementary grades, or is it subject to learning and specialization in the course of schooling, as part of the domain-specificity definition of the cortical neurological network of reading saccades?

### 2.4 The precursors of our experimental paradigm

As anticipated in the *Contribution* section, our experimental paradigm aims to maintain the ecological proximity to the natural reading task, primarily done aloud in the early elementary school grades. At the same time, the proposed experimental paradigm aims to minimize the burden of lexical access by using digits because digits are recognized rapidly and in an automated fashion from an early age (e.g., Åvall et al., 2019; Protopapas, 2023). A further objective is to maximize the accuracy in evaluating the proactive/predictive localization span. With these purposes in mind, we designed a battery of Automated Rapid Naming (RAN) serial tasks.

#### 2.4.1 RAN and visual scanning hypothesis

The Rapid Automatized Naming (RAN) task requires the subject to name familiar items (e.g., colors, objects, letters, or digits; Denckla, 1972; Denckla and Rudel, 1976) as quickly as possible. Literature has repeatedly shown that RAN can predict the subsequent development of reading ability both in transparent (e.g., Lervåg et al., 2009) and opaque spellings (e.g., Wolf and Bowers, 1999; Georgiou et al., 2008). Uncertain and contradictory results come from studies that have tried to demonstrate the RAN ability to predict spelling abilities (see Furnes and Samuelsson, 2011, for a review).

Some authors consider the RAN ability as a kind of microcircuitry within the broader reading circuitry and that the former is responsible for reading fluency in all orthographies (e.g., Norton and Wolf, 2012). Proponents of the dual-deficit theory of dyslexia argue that there are two possible disabilities, phonological and naming speed (e.g., Wolf and Bowers, 1999), and that RAN detects the latter. Thus, in that case, the RAN test would highlight mainly extraphonolgical disabilities.

The extraphonological aspects of the RAN test are investigated in Protopapas et al. (2013). The authors propose an inverse-reading variant of the classical RAN task, named Serial Backward Naming Task (SBNT). Subjects’ performance on the classical and inverse RAN variants are compared. Analyses show that the inverse RAN test successfully explains 49% of eye-movement variance during word reading and 12% of the variance in passage reading. Instead, the variance explained by the classical RAN test was 37 and 7%, respectively. Given the incongruence of SBNT with oculomotor computation and attentional unfolding typical of reading (see, e.g., Reichle et al., 1998, 2003; Engbert et al., 2005), this unexpected result reveals an inconsistency of the visual scanning hypothesis. Indeed, the visual scanning hypothesis postulates that the link between reading ability and serial RAN is the performance of sequential saccades and fixations in the normal reading direction, as proposed by Kuperman and colleagues (e.g., Kuperman and Dyke, 2011; Kuperman et al., 2016).

Henry et al. (2018) compare the classical and backward RAN tests by considering several novel item collections and tasks. The study’s results confirm the previously achieved by Protopapas et al. (2013). However, Henry et al. (2018) show that the visual scan hypothesis holds in both the classical and backward RAN variants. Indeed, the authors found better predictability in the classical RAN variant when the subjects read silently (without articulation). Their interpretation is that the eye-voice span (see, e.g., Inhoff et al., 2011; Norton and Wolf, 2012) in the verbalized version may have masked the advantages produced by the parafoveal preview benefit (see, e.g., Vasilev and Angele, 2017). In addition, their unnameable versions of the RAN (direct and inverse, which they call “pure oculomotor” RAN) explain a substantial and reliable amount of variance (10%) of the eye movements during passage reading.

#### 2.4.2 Errors and self-corrections

The eye-tracking studies mentioned above aim to understand the extent to which attentional and oculomotor components constitute the link between reading and RAN. Thus, albeit considering backward and non-verbal RAN variants, the authors do not propose variants where items are placed differently than the grid-like structure of the classical RAN test. Also, the authors decided not to consider the quantitative impact of specific errors made by the subjects (e.g., omissions, repetitions, verbal label errors), according to the original choice made by Denckla (1972) and kept in Wolf and Denckla (2005) and Wagner et al. (1999). This choice was motivated by the belief that specific errors and self-corrections would not increase the predictability of future reading ability. Even psychometric tests of optometric origin, such as Pierce (1972), King (1976), and Garzia and Richman (1987), do not leverage errors for diagnostic purposes. For instance, the Developmental Eye Movement (DEM) test—detailed in the following paragraph—uses errors exclusively to adjust running time measurements and does not consider self-corrections. Parallel to the studies of Denckla (Denckla, 1972), within the optometric clinic of developmental disorders of oculomotor reading skills, psychometric tests addressing the ability to read digits rapidly and accurately have been developed intending to identify dysfunctions of the saccadic oculomotor computation (see, Pierce, 1972; King, 1976).

#### 2.4.3 The DEM test

In 1987, Garzia and Richman (1987) introduced the DEM test (see also Garzia et al., 1990; Facchin, 2021) with the aim to disambiguate the role of rapid-naming and oculomotor skills in a serial digit RAN. Two subtests, A and B, where digits are arranged in vertical columns, are leveraged to verify the baseline performance attributed to phonological rapid naming skills. In contrast, a third subtest (named subtest C) involving only horizontal and carriage return saccades is devised to evaluate the additional weight of oculomotor computation. Therefore, the authors do not address the weight of the vertical oculomotor computation present in the two first subtests. However, the finding that the calculation of vertical saccades in people with dyslexia seems altered (longer saccadic latencies, Tiadi et al., 2014) calls into question the oculomotor neutrality of subtests A and B. The DEM Test (Garzia and Richman, 1987; Richman, 2009) has been criticized. On the one hand, the validity and reliability of the DEM test were questioned (Rouse et al., 2004; Orlansky et al., 2011). These criticisms were further discussed and addressed in Facchin and Maffioletti (2011) and Tassinari and DeLand (2005). On the other hand, the relationship between DEM and oculomotor reading abilities was highly criticized (Ayton et al., 2009). In particular, Ayton et al. (2009) conclude that performance on the DEM test does not correlate with horizontal saccadic eye-movement ability or symptomatology, even though it is related to reading performance and visual processing speed. However, the most destructive criticism of the logical foundations of the DEM test comes from the results produced by the studies by Facchin et al. (2014) on the “rotated DEM test,” which have led to the uncrowded equidistant horizontal arrangement of digits being considered easier, faster and more automated than vertical arrangement. Thus, subtests A and B lose their validity entirely as representatives of pure phonological skills in this new setting.

### 2.5 Our standpoint

For the reasons mentioned above, the purposes of ascertaining the development/evolution of the proactive/predictive localization span and clinical-ecological considerations, we decided not to design a “phonological-only” serial RAN, that is, without the contribution of oculomotor, attentional, and executive processes. In designing the individual subtests, we kept in mind the purpose of testing the extreme right collinear part of the span we mentioned in the part of the Review section devoted to word length span. In addition to differentiating the distance between targets (as already attempted by optometry-derived tests), we also introduced different tasks, distractors of various types, and one- or two-dimensional crowding conditions (see, e.g., Whitney and Levi, 2011; Stewart et al., 2020). We have also tried to keep the weight of the Phonological Awareness (PA) low by using digits as an item. Finally, based on these considerations and resolutions, we designed a multi-objective visuospatial test of attentional and executive oculomotor skills founded on a serial digit RAN task named Top-RAN (Topological Rapid Automatized Naming). The name Top-RAN highlights the crucial role of the spatial distribution (i.e., inter-distance, crowding) of targets (and sometimes distractors) in each subtest.

## 3 Methods

### 3.1 Protocol and motivations

We aim to highlight the extra-phonological processes of a serial digit RAN task (Wolf and Bowers, 1999; Wolf and Denckla, 2005) and, in particular, those processes related to attentional and saccadic abilities (Protopapas et al., 2013; Henry et al., 2018). We devised several RAN tasks comprising visuospatial difficulties, distractors, and crowding to do this.

Traditionally, naming errors (omissions, repetitions, misspellings) and self-corrections have been considered unpredictive of future reading skills (see, e.g., Denckla, 1972; Wagner et al., 1999; Wolf and Denckla, 2005). The proposed test battery aims to understand and measure how an increase in visuospatial difficulty impacts performance and errors in frequency and type. Specifically, the Top-RAN test battery aims to: (1) Understand whether the subtests can effectively discriminate subjects by age and school period. (2) Understand whether subtests discriminate subjects based on Phonological Awareness and Language Processing, given the presence of non-native Italian-speaking pupils. (3) Understand whether visuospatial alterations in the Top-RAN (differential target distances, presence of crowding or distractors, task differences) can produce significant differences in performance between subtests. (4) We want to investigate whether different tasks result in specific errors. (5) Understand whether differences between subtests, in terms of errors, corrections, and execution time, gradually diminish as age and school period progress or remain stable over time. (6) Finally, understand whether visuospatial alterations (differential target distances, presence of crowding or distractors, task differences) in a digit-RAN can cause errors and self-corrections discriminating subjects’ performance.

In the following sections, we discuss the proposed subtests, the dataset structure, and the data-analysis pipelines employed to achieve the aims listed above.

### 3.2 Transparency and openness

Before beginning the experimental design and data collection, it was imperative to obtain authorization from the Didactic Directorate of each institute mentioned in the manuscript, as per the current regulations of the Italian National Association of Psychologists. Each Institute’s School Council should have informed the teachers about the screening protocol and obtained their consent before informing the children’s parents about the possibility of participating in the screening. Teachers who expressed a favorable opinion were then tasked with presenting the objectives of the screening investigation to the parents, who would then provide their informed consent. The informed consent document should have described the type of tests and methods used during the screening, as well as the procedures involved.

We are also committed to making our data and materials available upon request to facilitate further research and promote open scientific inquiry. Our dataset data can be accessed at link https://github.com/LimenResearch/topran_stats/tree/main/data, whereas the computer code and methods required to replicate the analyses are available at https://github.com/LimenResearch/topran_stats.

### 3.3 The Top-RAN test battery

The Top-RAN battery comprises 16 subtests that differ on the task, distractors, and crowding elements. The tasks are pseudo, vertical, and inverted reading, jumps between columns, and serpentine paths. Additional elements that differentiate the subtests from each other are listed in Table 1.

**Table 1:** Tasks, and distractor, crowding structure per subtest.

Test	Task	Distractor/Crowding
1	Pseudo-reading	–
2	Pseudo-reading	Horizontal Crowding
3	Pseudo-reading	–
4	Pseudo-reading	Antigrouping
5	Pseudo-reading	Antigrouping/Distribuited Salience
5b	Pseudo-reading	Oblique Antigrouping
6	Vertical Reading	Vertical Crowding
7a	Jumps between columns	–
7b	Jumps between columns	Attenuated Bidimensional Crowding
8a	Jumps between columns	Bidimensional Crowding
8b	Jumps between columns	Bidimensional Crowding
9	Serpentine Path	–
10	Serpentine Path	Bidimensional Crowding
11	Inverted Reading	–
12	Inverted Reading	Antigrouping
13	Inverted Reading	Antigrouping/Distribuited Salience

The primary purpose of the subtests was to highlight, through the increase of difficulty, the voluntary components of oculomotor behavior (components that, in other paradigms, have shown developmental trends. See, e.g., antisaccade literature; Biscaldi et al., 2000; Munoz and Everling, 2004; Montez et al., 2019; van Ede et al., 2020). However, in the proposed protocol, we leverage a static paradigm, i.e., without moving elements or abrupt onset. This choice aims to produce an ecological and perceptual closeness with regular reading. The practical features of Top-RAN that bring it ecologically closer to reading are as follows: (1) Saccadic computation is aimed at the acquisition of visual information and not at the simple localization of meaningless objects (as is the case in experimental paradigms from the laboratory, e.g., prosaccade). (2) The subject reads aloud, a familiar practice in the first two elementary classes, as well as in all RAN paradigms. (3) The task set pre-defines the seriality of voluntary gaze shift in much the same way as in reading because: (a) it requires the performance of progressive left-to-right saccades [#1-2-3-4-5-7a and b-8a and b-9-10], or regressions typical of right-to-left rereading (9-10-11-12-13), or top-down saccades as in Chinese spelling (6-9-10). (b) In all but two of the Sub-tests (9-10), the performance of saccadic back-to-back saccades is required. (c) The saccadic targets (digits) to be named are not entirely predictable because the subject, whenever he moves his/her eyes in the predetermined order of the task, never knows what the following digits to be acquired and named will be (for the limitations of this statement, see Section 3.4.2 Familiarity with the task and effects on automaticity). (4) the subject has almost unlimited freedom of head movement, allowing for a much more natural saccade-VOR alternation that corresponds to the subject’s habitual behavior (Leigh and Zee David, 1999). (5) The use of paper materials to present subtests ensures the presence of a black-and-white contrast typical of the school environment. (6) Given the subjects’ age, the absence of intrusive measuring instruments excludes sources of anxiety, distraction, or an altered relationship with the examiner.

We devised five subtest groups organized in sequence from the most straightforward subtest (with minimized impact of distractors or crowding) to the most complex (with maximum impact of distractor or crowding).

#### 3.3.1 One-dimensional crowding

This category comprises subtest 1 (minimum crowding), subtest 2 (horizontal crowding), and subtest 6 (vertical crowding). These tests aim to capture the evolution in handling a crowded reading environment. In this context, the word *handle* means minimizing errors and self-corrections while keeping execution times low.

#### 3.3.2 Antigrouping

While subjects are trained to handle horizontal word grouping, this category of subtests requires the subject to perform voluntary horizontal saccades (by rows) along targets arranged in vertical (serpentine columns) or oblique groupings. For this reason and by association with the antisaccade paradigm (Hallett, 1978), we named this subtest category *antigrouping*. This category comprises subtests 3 (neutralized antigrouping), 4 (vertical antigrouping for proximity), 5 (vertical antigrouping for color, area, and proximity), and 5b (oblique area antigrouping). The goal of antigrouping is to obtain a test that, instead of producing an eye movement in the direction spatially opposite to the distractor’s onset (antisaccade task – inversion of interfering dynamic component), requires horizontal voluntary eye movements in contrast to vertical proximity grouping (Brooks, 2015) of digits arranged in serpentine columns (inversion of interfering static component). In subtest 5b, in addition to grouping in vertical serpentine columns, there are groupings by common region on oblique strips.

#### 3.3.3 Alternate columns in two-dimensional crowding

7a (minimum crowding), 7b (attenuated crowding effect/grid effect), 8a and 8b (two-dimensional crowding). These paradigms are derived and adapted from the practice of Hart Charts in Optometric Vision Therapy (Scheiman and Wick, 2019).

#### 3.3.4 Serpentine paths in two-dimensional crowding

9 (no crowding around the path) and 10 (two-dimensional crowding around the path). The arrangement of target digits involves sudden changes in the path with 90 turns, down, right, or left.

#### 3.3.5 Antigrouping, reverse read

11 (neutralized antigrouping), 12 (vertical proximity antigrouping), and 13 (vertical proximity, color, and area antigrouping). These subtests are directly derived from subtests 3, 4, and 5. However, they require the subject to read from the bottom rightmost digit, proceeding from right to left.

We designed the subtests in Adobe Illustrator. Subtests were printed with the Epson ET-7750 inkjet printer at a maximum color resolution of 5760 × 1440 dpi on Epson Matte Paper Heavy Weight A4 sheets (167 g/m) with matte lamination (Fellows Matt thickness 125 m) and presented on an almost vertical lectern (85) at 33 cm from the subjects. Subtests 1, 2, 3, 4, 5, 6, 7a, 7b, 8a, 8b, 9, and 10 were administered to all classes and in all considered school periods. Initial analyses of the data from administration in the first two school periods (1st-grade third trimester and 2nd-grade first trimester: hereafter 1B and 2A) showed a marked reduction in the errors pronounced during the subtests with antigrouping. This trend predicted a rapid overcoming of the difficulty produced by this paradigm. Accordingly, we designed subtests 5b, 11, 12, and 13. In particular, we built subtest 5b based on the frequency of Channeled LC (Lane Change: see Supplemental materials) errors in subtests 4 and 5. Subtests 5b, 11, 12, and 13 were administered only from the 2nd-grade third trimester (2B).

Subtests present different amounts of items: 80 for subtests 1, 2, 3, 4, 5, 5b, 6, 11, 12, 13; 20 for subtests 7a, 7b, 8a, 8b; and 88 for subtests 9 and 10. The different number of items could be a problem only in case of sustained visual attention difficulties (Skogsberg et al., 2015). However, other RAN tests have traditionally used very different amounts of items. The RAN/RAS test (Wolf and Denckla, 2005) includes 50 items (5 rows of 10 items); the CTOPP (Wagner et al., 1999) provides two pages of 36 items each (4 lines x 9 items) for a total of 72 items. The RAN Santa Lucia (Luca et al., 2005) consists of 2 pages, 50 items each (10 lines x 5 items) of 100 items. Supplementary Table S1 shows a summary of the contents of the individual subtests.

### 3.4 Prolonged and repeated use of the digital RAN – problems and advantages

#### 3.4.1 Number recognition speed and numerical processing ability

The literature reveals a dynamic and evolving understanding of the dissociation between digit naming skills and possession of quantity, ordinality, and cardinal concepts of numbers. This is not a static field of study, but a vibrant and ever-changing research landscape. For example, Shaki et al. (2009) found that verbal naming tasks are not sensitive to the SNARC (Spatial-Numerical Association of Response Codes) effect, indicating a dissociation between verbal naming and numerical processing. More recently, Yuan et al. (2019) have added a new layer of understanding, emphasizing the importance of children’s ability to determine the exact quantity of small sets and set names. The existence of specific brain areas (e.g., differential contribution of the left and right inferior parietal lobules to number processing; Chochon et al., 1999) dedicated to processing in mathematics, separate from linguistic processing, has also been underscored by neuropsychological case studies demonstrating a dissociation between number naming and number comprehension (Fias et al., 2001). This dynamic nature of the field is further exemplified by the research of Jun-Hui et al. (2018), which has indicated that the brain regions involved in mathematical computations change with age and training, further supporting the notion of specialized brain activation for mathematical processing that is progressively enriched with semantic content that it does not initially possess. In dyslexia studies, the critical importance of the dissociation between print-to-sound conversion and phonological representation has been emphasized, further supporting the dissociation between different aspects of number processing (Colomé and Noël, 2012).

While the naming-semantic dissociation of numbers seems to be generically accepted, especially in the early stages of schooling, it has been suggested that automated rapid naming of digits is a strong predictor of reading ability in both typical and deficit readers (Jones et al., 2009; Hornung et al., 2017). This is supported by the finding that digit naming is faster than other stimuli, indicating a greater degree of automaticity in digit naming (Pan et al., 2013). Also, Protopapas (2023) recently confirmed the superiority of serial automated rapid digit naming (detected in early elementary grades) in predicting future reading skills.

#### 3.4.2 Familiarity with the task and its effects on automaticity

One possible alteration in performance may come from long-term memorization of the digit sequence when it is repeated identically (as in tasks 3–4-5-5b-11-12-13). However, the automaticity of digit naming may be increased (and not reduced) precisely in its linguistic component (lexical access to the verbal label attributed in long-term memory to the grapheme) by the possible memorization of the sequence of numbers. In other words, since the task was intended to assess exclusively the visuospatial effects brought about by the arrangement of digits and distractors/crowding on the sheet, the existence of verbal forms of memory of the number sequence would have increased the speed of retrieval of the verbal label of the single digit thereby enhancing its automaticity. Thus, slowdowns or errors in exercises with identical sequences but different visuospatial difficulties would have further confirmed the existence of specific levels of visuospatial difficulties (this happens mainly in tasks 3-4-5-5b-11-12-13). The possible existence of this memorization (especially likely in HPC or gifted subjects) in the case of the finding of worse performance (more remarkable slowness and more significant errors) in tasks with higher predicted visuospatial difficulty, at the limit may have the effect of having caused the differences in performance that existed between tests to be underestimated (rather than overestimated). For this reason, the performance differences recorded with increasing difficulty (particularly from subtest 3 to 5b and from subtest 11 to 13) can only increase the validity of our results.

In this regard, we were comforted by the literature regarding measurements of eye movements obtained by rereading the same text: the only significant effect was the reduction in the proportion of regressions performed (Schnitzer and Kowler, 2006). However, only visuoperceptive-based regressions of 1–2 characters were reduced, and not those greater than four characters (taking into account that the re-reading regressions used to optimize text comprehension do not exceed 10 characters, this effect did not occur on a linguistic basis either). In contrast, the clustering of spatial localizations of fixations on words was highly repeatable, which was found to be related to local text features rather than individual differences pertaining to mean saccade length (Schnitzer and Kowler, 2006). In the context of our study, these results led us not to consider repeating the same digits in very similar locations from subtest to subtest as capable of significant visuospatial impact. Instead, we expected more substantial effects of grouping by proximity (antigrouping) and grouping by color and area (salience). However, this “spatial” and “verbal” repetition of digits may have further facilitated automated naming, increasing the validity of results on differences in performance between subtests. The latter consideration does not exclude the possibility that in higher school grades (fourth and fifth), this repetition may have obscured visuospatial effects that are otherwise present.

### 3.5 Participants

In Table 2, we report the composition of our sample of subjects. All schools participated in the longitudinal project, except for Mirabello School, which contributed only one class (3^rd^-grade third trimester).

**Table 2:** Participants.

	All schools	Non-native	Ada Negri	S. Damiano Piemont	Mirabello 3G3T	Lombardy
Total	142	46	55 (2018 n = 39; 2019 n = 16)	66 (2018 n = 47; 2019 n = 19)	21	76
M	66	21	27	29	10	37
F	76	25	28	37	11	39

In total, we performed 526 administrations of the Top-RAN battery, of which 250 in the reduced version (consisting of 12 subtests, administered up to and including the 2nd-grade first trimester) and 276 in the full version (consisting of 16 subtests, administered starting from the 2nd-grade third trimester inclusive). The scholastic phases of administration were as follows: 1B, 2A, 2B, 3A, 3B, 4A, 4B, and 5B (see Supplementary material). We established the following inclusion criteria: automated recognition of one-digit numbers 0 to 9 and visual acuity of not less than 0.32 decimals. For the first criterion, each participant underwent the BIN 4–6© (Molin et al., 2007) test; for the second criterion, each participant underwent the Precision Vision LEA Symbols© Visual Acuity test at 16 inches (cat. no. 2508, developed by Lea Hyvärinen, M.D.). Following these criteria, we excluded no subjects. All pupils’ parents have given their written consent to the children’s participation in the project.

### 3.6 Procedure

The subject sat in front of the lectern at an initial distance of 33 cm (measured as the segment connecting the nasal root of the subject to the center of mass of the lectern). In case of insufficient ambient lighting, sheets were illuminated with an LED lamp mounted on the lectern. As with previous oculomotor psychometric tests (Pierce, 1972; King, 1976; Garzia and Richman, 1987), we chose not to limit the movements of the subject’s head through a forehead rest. Thus, the subject could freely move closer, up to 25 cm to the sheet. The digits printed in Courier font, at a distance of 33 cm subtended a visual angle of 0,557° vertically and 0,352° horizontally; at 25 cm, 0,736° vertically and 0,465° horizontally. At the distance of 25 cm, only the targets distant 8-character spaces from each other (present in subtests 3, 4, 5, and 5b: 7 char/space = 4,755°; 8 char/space = 5,434°) exceeded the limits of the parafoveal area (≈ 5°) by eccentricity. The subjects moved closer than 25 cm from the lectern only in 76 out of 8,416 subtest administrations, i.e., mainly for students attending the first-grade third trimester and second-grade (1st trimester).

In all subtests, the subjects were required to avoid keeping the sign by pointing with their fingers. This request stems from the fact that the proposed test aims to verify the ability of overt attention orientation without motor integration. The intertwining of visual attention and motor integration would add confounds to be considered during the analyses of the collected data. Furthermore, using pointing can result in undesirable performance improvement (or impairment) (Neggers and Bekkering, 2000): e.g., we do not know the effect of pointing on the active management of crowding. As a result, we could not formulate grounded hypotheses on the reading times, number of errors, and self-correction trends.

The novelty of some paradigms (e.g., antigrouping, alternate columns reading, serpentine paths) required students to read by following unconventional spatial constraints, especially in the case of first-grade students. Thus, we have planned for the gradual increase in the difficulty level to serve as perceptual-oculomotor valuable priming for a better task introduction. Therefore, we intended priming, verbal explanations, and pointing of the administrator to ensure that subjects understood the task. In case of substantially incorrect comprehension (e.g., use of bustrophedic reading), we stopped the subject, repeating the explanation and the whole subtest.

In Lombardy schools (Mirabello; Ada Negri), we recorded the running times using a stopwatch and manually transcribed the errors onto prepared scoresheets. In the San Damiano school, we recorded children’s voices with a smartphone app (HT Recorder), calculated the running times, and transcribed the errors on scoresheets. After this transcription, the data was entered into a custom-designed Claris FileMaker database, classifying errors.

### 3.7 Variables

Variables shared by all subtests are listed in Table 3. Instead, Table 4 showcases all variables recorded for a specific subset of subtests. Figures 1A–D show examples of errors and self-corrections. See the Supplementary material for details.

**Table 3:** Variables shared by all subtests.

Time variables	Omissions	Additions	Self-corrections	Other
Time	Total Omission Errors	Total Addition Errors	Total Self-corrections	Orders Errors
Average time per digit		*In loco* Re-readings	Self-correction of Label Errors	Addition-Subtraction Balance
		Secondary *In loco* Re-readings	Self-correction of Omission Errors	Unsolvable sign loss
		Secondary Post-regression Re-readings		Finger Pointing
		Non-secondary Post-regression Re-readings		

**Table 4:** Variables associated with specific subsets of tests.

Errors/Self-corrections	Subtests
*Lane Change errors* (LC), their subcategories, and their self-corrections	3,4, 5, 5b, 7a, 7b, 8a, 8b, 11, 12, 13
*Carriage Return errors* (CR), their subcategories, and their self-corrections	3,4, 5, 5b, 7a, 7b, 8a, 8b, 11, 12, 13
*Column errors* and their self-corrections	7b, 8a, 8b
*Path errors* (Off-path) and their self-corrections	9, 10
*Second transit Re-readings*	3, 4, 5, 5b, 7a, 7b, 8a, 8b, 10, 11, 12, 13
*Non-secondary omission errors*	3, 4, 5, 5b, 7a, 7b, 8a, 8b, 10, 11, 12, 13
*Channeled LCs*, *Anomalous LCs*, their self-corrections, and *Visuospatial Memory CRs*	5b

### 3.8 Analysis pipelines

#### 3.8.1 Aim

With in mind the goals listed in Protocol and Motivations, we devised data-analysis pipelines addressing the following questions: (a) *Expressiveness*. Can Top-RAN retrieve individuality in a possibly homogeneous population? (b) *Fairness*. Can the subjects’ information be retrieved from their performance? (c) *Explainability*. Is it possible to map subtests to an informative, intelligible space and can we measure distances between subtests?

#### 3.8.2 Implementation

To address these questions, we devised Top-RANStats, an open-source Python package that implements all the data-analysis pipelines in this manuscript. Top-RANStats is available at: https://github.com/LimenResearch/topran_stats, and it is designed to allow for swift implementation of fairly general statistical and machine learning pipelines tackling the quantitative and qualitative aspects related to the questions mentioned above. As an example, by leveraging the data-exploration tools comprised in Top-RANStats, we generated the histograms in Figure 1E. There, we represent some key features of the proposed dataset.

#### 3.8.3 Algorithms

**Figure 2:**
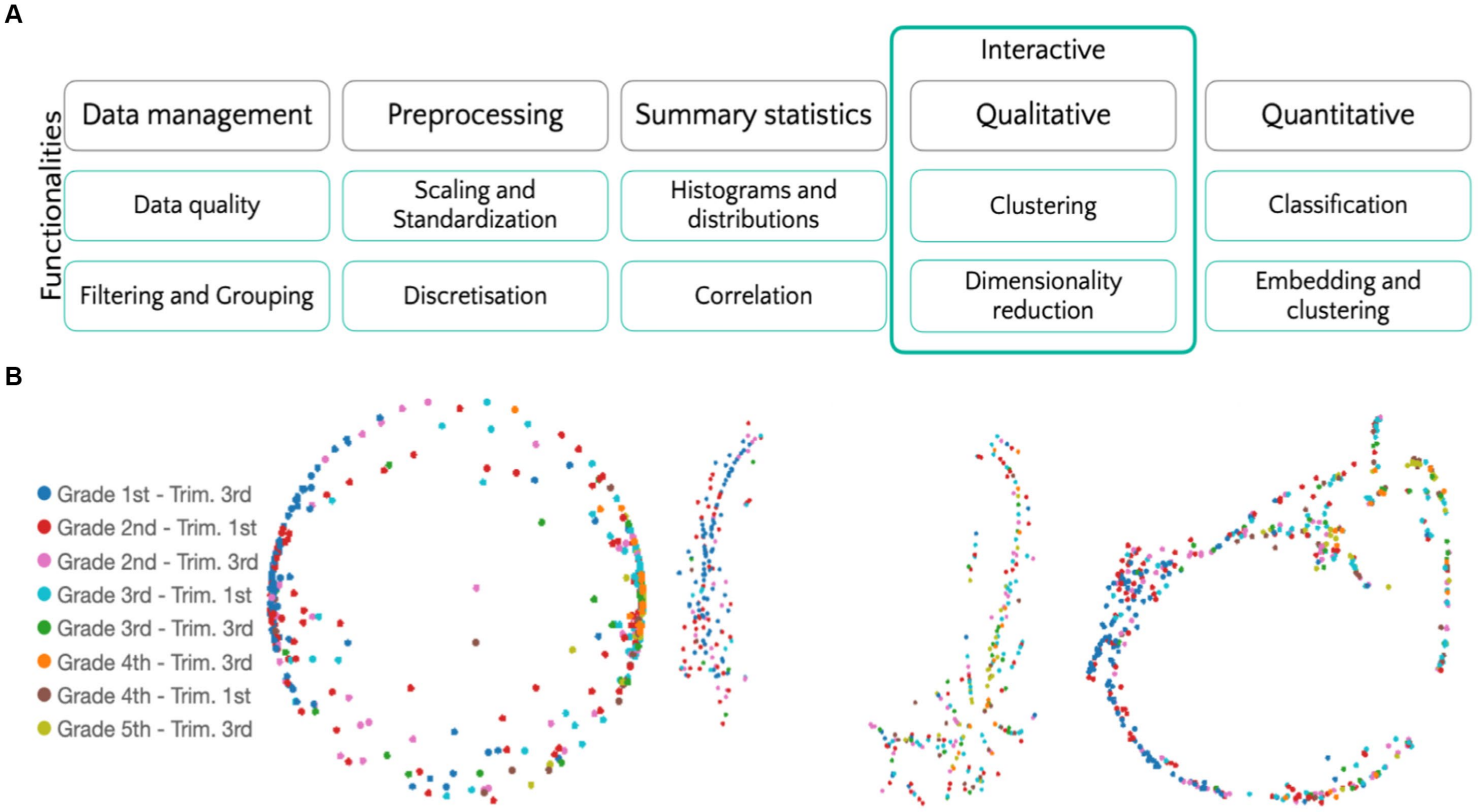
Analysis pipelines and data exploration. **(A)** The structure of the analysis Python library we implemented and utilized. **(B)** From left to right: PCA, UMAP and t-SNE computed on the entire dataset (time, errors, and self-corrections) and labeled according to the students’ scholastic phase.

Top-RANStats is organized into four modules, as showcased in Figure 2A. Data management, preprocessing, and summary statistics modules are designed to read, normalize, and check the quality of our data. The data analysis modules address two distinct data-analytical aspects. On the one hand, Top-RANStats implements a qualitative analysis suite for clustering, dimensionality, and cardinality reduction methods. These algorithms allow the users to explore their datasets via interactive visualizations. On the other hand, on the quantitative side, we leverage classical kernel-based classification methods and hierarchical clustering for measuring distances across subgroups of students and subtests.

In the following paragraphs, we provide a brief description of the algorithms employed to address the points listed above. For further details, we refer the reader to Supplementary material.

#### 3.8.4 Expressiveness

**Figure 3:**
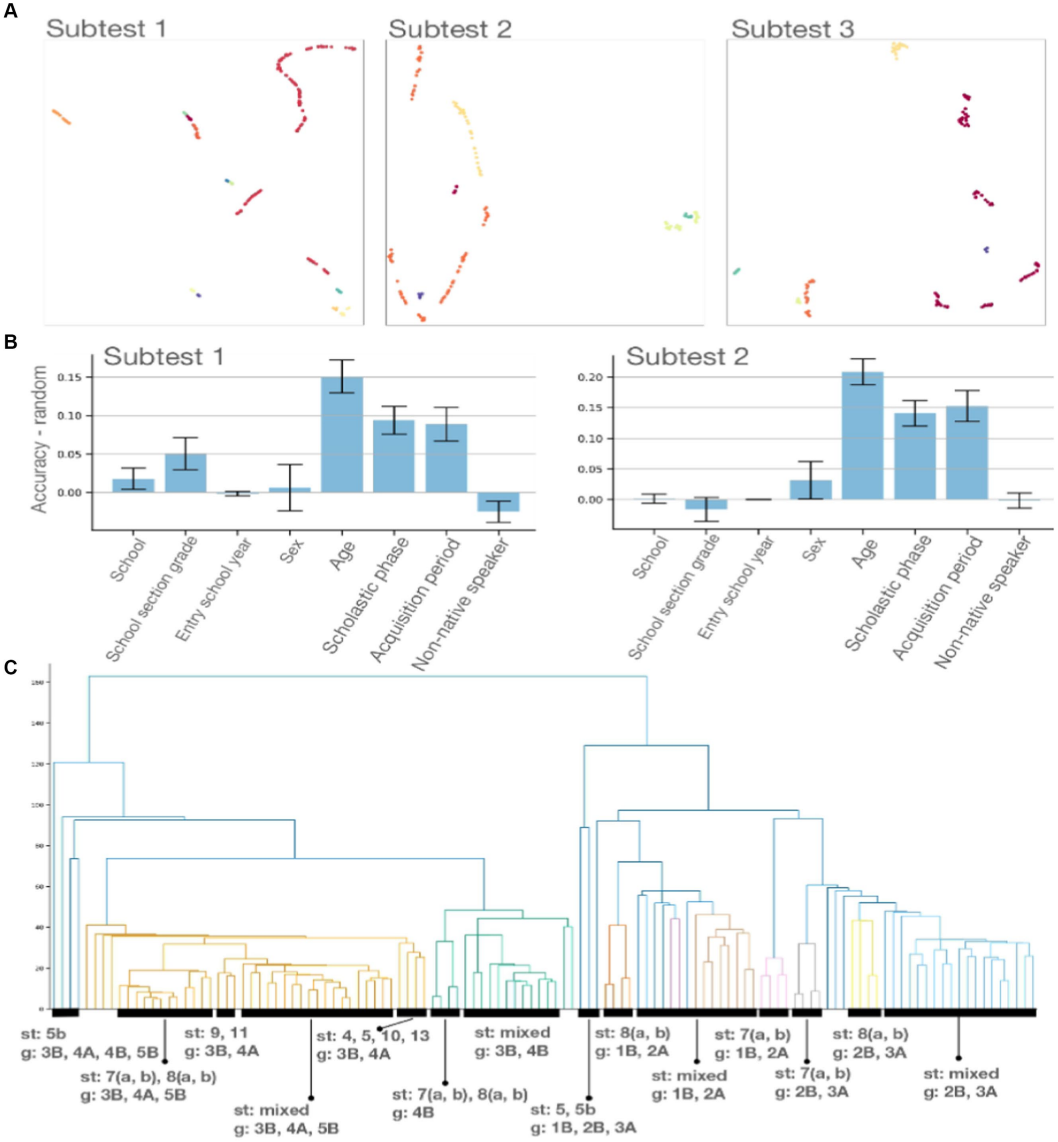
**(A)** Students’ performance clustering by subtest. Point are color coded according to the cluster identified by DBScan. Noisy points have been removed. Axes are arbitrary units as a result if applying UMAP to reduce the dimensionality of error counts associated with each student to two dimension. **(B)** Bias detection. We leverage a Support Vector Classifier fitted on 70% of the data and report performance on the remaining 30% (test set). Error bars are obtained by evaluating the performance of the model on random labeling of the test set. **(C)** Subtests hierarchical clustering. Distances are computed by considering the summary statistics of the error counts associated with each subtest.

Data exploration and swift interpretation, even for the users unfamiliar with data analysis is a crucial step for formulating data-driven hypotheses and gaining insight into the information carried or lacking in a specific dataset. We realized scripts to easily apply standard dimensionality reduction techniques (namely, PCA, t-SNE, and UMAP, see Supplementary material for details and intuitions) to tabular data. Moreover, we integrated Tensorboard into our pipelines. This integration makes it possible not only to visualize but even explore high-dimensional data interactively. As an example, in Figure 2B, we normalize the error data relative to subtest 1 and compute the projections associated with the three dimensionality-reduction algorithms listed above. Data can easily be color coded according to the students’ attribute—Scholastic Phase in the figure. Moreover, we implement a clustering method allowing to: (1) Adopt a variety of clustering methods; (2) Visualize the resulting clustering in low dimension; (3) Exclude noisy points. Cluster computed per subtest through this custom method can be easily visualized as interactive, web-based plots showing only significant (non-noisy) points, color coded according to their cluster (see Figure 3A).

#### 3.8.5 Fairness

We utilize the information available for each student—School, School section grade, Entry school year, Sex, Age, Scholastic phase, Acquisition period, and Non-native speaker columns in the Top-RAN dataset (see Supplementary material for details)—to detect possible subtest biases. To do this, first, we split the data randomly in half to obtain the train and subtest sets. Then, we fit a Support Vector Classifier (SVC)—see Supplementary material for details—with a radial basis function kernel on the train set and produce predictions on the subtest set for each of the labeling functions listed above. Finally, we compute the difference between the classification accuracy obtained by the SVC on the correctly labeled subtest set with the accuracy obtained by the same models on random labeling of the same subtest set.

Remark—Age is the only continuous feature acquired per student. Classification of this feature relies on a discretization such that the same number of points is assigned to each bin.

Figure 3B shows the resulting barplots for subtests 1 and 2. We consider a subtest to be biased with respect to a feature if the accuracy difference is significantly greater than 0.

#### 3.8.6 Explainability

Inspired by the molecular clock hypothesis (Thorpe, 1982), we aim to map samples grouped by subtest to a meaningful mathematical space endowed with a well-defined notion of distance. The idea, detailed in Supplementary material, consists in computing summary statistics based on the students’ performance per subtest, thus associating a feature vector $F_{i}=\left(f_{i,1},\ldots ,f_{i,n}\right)$ to the i th subtest. We then interpret these feature vectors as point in the Euclidean space and use the Euclidean metric to measure distances between them. This notion of distance allows us to compute the subtests 15 hierarchical clustering and thus a dendrogram as the one represented in Figure 3C.

## 4 Results

### 4.1 Expressiveness

Data acquired through the Top-RAN test consists of a collection of discrete columns counting several types of error, self-corrections, and other behavioral components. Additionally, three continuous columns account for the age of the students, and the time (average and per-digit) students took to complete each subtest (see Participants subsection and Supplementary material). The information carried by a limited number of counters, even fewer continuous variables, for a population counting few hundreds of samples, can potentially be fully described by noisy distributions, or give rise to indistinguishable clusterings.

Our computational experiments aims to prove that subtests can reliably detect subpopulations on a subtest basis, and discard noisy points. Figure 3A shows clusters drawn from the three first subtests. Clusters were computed via the DBScan algorithm (Schubert et al., 2017) with min_samples =5 and eps=0.5 . The visualization is realized through the UMAP algorithm (see Supplementary material) with parameters n_neighbors =15 , n_components =2 , and Euclidean metric. We can observe how the clustering algorithm identified non-noisy, distinct subpopulations for each subtest. An interactive visualization for all subtests are available in https://github.com/LimenResearch/topran_stats/reports/clustering.

Remark—The clustering algorithm we leveraged in our experiment is unsupervised. UMAP – the algorithm we utilize to render high-dimensional data in a two-dimensional space – is also unsupervised.

### 4.2 Fairness

We utilize general information (e.g., age, sex and native language), and scholastic data (e.g., school, section, grade) to evaluate the presence of possible biases introduced by each subtest. Bias detection is done via the algorithm described in Analysis pipelines – Fairness.

We observe that Age of Administration, School Phase, and Survey period can be regressed from students’ performance in all subtests. Performance predicts successfully the students’ school for subtest 5 and predicts with a significant accuracy above random the school + section + year column (i.e., class and teacher impact) for subtests 5, 5b, and 9. However, predictions are less significant for subtests 7a, 3, 8a, 1, 6, and 8b. Performance predicts only marginally students’ Entry School Year for subtest 5. The performance successfully predicts the students’ sex for subtest 13 and only marginally for subtests 5b, 7a, and 7b. Table 5 lists all detected biases.

**Table 5:** Bias detection and quantification.

Subest	% a.r.	$\sigma \cdot 10^{2}$	Label	Subest	% a.r.	$\sigma \cdot 10^{2}$	Label
01	13.5	2.2	Age	07b	15.0	1.9	Age
01	16.4	2.1	SP	07b	11.1	2.1	SP
01	9.9	2.6	AP	07b	6.5	2.1	Sex
02	16.7	2.2	Age	08a	16.0	2.3	Age
02	16.9	2.2	SP	08a	10.7	2.4	SP
02	9.3	2.0	AP	08a	5.2	2.0	AP
03	12.4	2.0	Age	08b	12.6	2.0	Age
03	10.1	2.1	SP	08b	11.2	2.3	SP
03	5.4	2.1	AP	08b	5.3	2.1	AP
04	10.1	2.3	Age	09	11.5	2.1	Age
04	13.6	2.2	SP	09	6.3	2.0	SP
04	12.9	2.3	AP	09	7.0	2.1	SSY
05	11.2	2.7	Age	10	14.4	2.2	Age
05	9.5	2.1	SP	10	10.1	2.2	SP
05	6.7	2.0	AP	10	8.6	2.3	AP
05	6.6	1.7	School	11	10.8	3.1	AP
05	7.1	2.2	SSY	11	9.6	3.2	Age
05b	10.7	3.0	Age	12	10.6	3.4	AP
05b	5.8	3.3	SP	12	8.1	2.8	SP
05b	7.7	3.0	SSY	12	8.1	3.1	Age
05b	7.3	3.0	Sex	13	11.1	3.4	Sex
06	12.6	2.2	Age	13	13.1	3.3	SP
06	12.4	2.2	SP	13	19.2	3.4	AP
06	11.0	2.4	AP	13	9.9	3.1	Age
07a	17.0	2.5	Age				
07a	10.4	2.1	SP				
07a	10.9	2.4	AP				
07a	7.5	3.0	Sex				

% a.r. is the difference between the classification accuracy on correctly labeled subtest data, against the average accuracy obtained by the same model on 100 random labeling. We list only values with accuracy score above random plus one standard deviation of the error. See Figure 2B. As possible biases, we considered School, School + Section + Year (SSY), School Year (Y), Sex, Age, Scholastic Phase (SP), Acquisition period (AP). For details see Section 3.3 and Supplementary material.

In summary, the scholastic phase and information concerning scholastic aspects such as school and specific teacher (section) play a crucial role in determining the student’s performance, highlighting the role played by active learning. Instead, the age bias partly proves the natural betterment due to physical growth.

The non-significant prediction accuracy of the Non-Native Speaker column from students’ performance indicates the almost nil impact of language in the administered subtests.

Remark—The performance reported qualitatively above and rendered quantitatively in Figure 3B are computed on the test dataset resulting from a 70% (train) / 30% (test) split of the dataset. Thus, reported accuracies above random indicate the model's ability – fitted to the training dataset and after convergence – to associate each error count to the correct label.

### 4.3 Explainability

From the analysis of the dendrogram in Figure 3C, it appears that differences between subtests exist and can distinguish ranges of school periods. Within these ranges, the differences between subtests seem to be: (a) More pronounced in the first range (1B/2A). (b) Less marked in the second range (2B/3B). (c) Even less marked in the 4B. (d) Even less marked in other school periods (3B/5B). Observations concerning the individual subtests are listed in Supplementary materials.

Analysis of the dendrogram in 3 panel (c) allows us to observe the following: (A) In the 3B/5B range, there is a sub-range for 4B school period (divided into two subsets: [8a 8b 7a 7b] and [Other subtests]). (B) 7A and 7b in the 1B/3A range are subtests that differ strongly from all the others as if they were outliers. Only in the next range (3B/5B) do they get closer to 8a and 8b until the grouping with the latter in 5B. (C) 8a and 8b group together and separate from other subtests in the 1B/2A range; they tend to do the same thing in the 2B/3A range; they maintain good separation from 7a and 7b and from the other subtests up to 5th grade.

#### 4.3.1 No-time dendrogram

From the analysis of no-time dendrogram (see Supplementary material), it appears that differences between subtests exist even if we exclude statistics regarding execution times, using only execution errors and self-corrections. However, they occur with the same trends as the dendrogram, including the time variables. Nevertheless, the no-time dendrogram differs from the complete dendrogram for the following characteristics: (a) A greater discriminative capacity between the individual school periods within the first two ranges. In fact, the dendrogram highlights four sub-ranges: 1B; 2A; 2B; 3A. (b) In the first two ranges, the 7a and 7b subtests remain segregated from the other subtests but are grouped by school period. (c) In the first two ranges, the 8a and 8b subtests remain segregated from the other subtests but are grouped by school period. (d) In the first two school periods, subtests 5, 4, 10, and 3 are particularly difficult and diversified. (e) Subtest 3 maintains its differentiation until 2B. (f) In the third and fourth sub-ranges (2B/3A), 5 and 13 subtests are highly differentiated from the others. (g) In the 3B/5B range, the differentiation between subtests arranges itself similarly to what happens in the dendrogram that includes time variables.

To sum up, the analysis of the collected data showed that: (A) The battery of tests was transparent concerning its biases (School, school year of first administration, native speaker) for each subtest and only marginally sensitive for some biases (sex seems to have weight only in subtests 6, 7b, and 13, and gives a more substantial contribution to the classification of subtests 10 and 11; The contribution to classification according to the School of belonging is marginal for subtests 1, 2, 4, 5, and 9, and is robust for subtest 8a). (B) The classification according to the different teams of teachers and classroom environments seems to be significant for 5, 11, 12, 9, 5b, and 4 but marginal for subtests 7a, 3, 8a, 1, 6, and 8b. (C) All subtests accurately classify individuals mainly by the age of administration, school stage, and administration period. (D) Visuospatial alterations of a serial RAN task of digits can produce differences between the various subtests concerning execution times and errors. (E) Unlike what happens in RAN literature, in our experimental paradigms, the impact of errors is not negligible: it becomes progressively so starting from the third grade last trimester (learning accuracy not explainable on phonological bases). Moreover, from the end of the first grade to the beginning of the 3^rd^ grade, errors can discriminate performance by school period better than an overall evaluation of performance (time variables + error variables). (F) The seven categories of error we excluded from the evaluation with dendrograms are the result and proof of the considerable differences among various subtests determined by tasks and spatial difficulties. For this reason, they will be the subject of future studies. (G) The error types included in dendrograms evaluation are common to all experimental paradigms used. Thus, they represent a constant joint basis throughout the development of the visuospatial skills involved.

The differences within the first sequence (One-dimensional Crowding) are minimal in all school periods. In the Supplementary materials, we enumerate and scrutinize the occurrences in other sequences, specifically addressing the dynamics of Antigrouping, Alternate Columns in Two-Dimensional Crowding, Serpentine Paths in Two-Dimensional Crowding, and Antigrouping Reverse Read.

Overall, we can say that out of the three scenarios we predicted, the third one has indeed occurred: visuospatial skills differ according to the task and evolve, producing not only a progressive and differentiated reduction in execution times but also an equally progressive and differentiated reduction in errors and self-corrections.

### 4.4 Summary

Given the complexity of our results, we summarize them below:

- The Top-RAN battery’s unique ability to identify sub-populations through its subtests, particularly by highlighting outlier subjects, underscores its potential for in-depth study and ignites the imagination for further research.
- Subtest performance appears to be highly influenced by active learning (*cf.* home school and specific teacher) and partially by the physical growth factor. However, within the limits of our knowledge, no specific teaching trains subjects in voluntary gaze orientation in the presence of different types of crowding and distractors and in the absence of the support of cueing or other means of reducing the impact of these sources of error. Therefore, we can hypothesize the presence of implicit learning of voluntary gaze orientation skills in schooling.
- The role of language processing in this type of learning seems less important, not only because of the use of digits as targets to be named (low demand for language processing) but also because subtests do not reliably discriminate individuals based on their language or ethnicity of origin.
- There are reliable differences in performance between subtests, indicating that different subtests can target different subcomponents of voluntary gaze orientation skills.
- The performance differences between the different subtests can reliably distinguish between different school periods of administration, especially the early school stages (1B, 2A, 2B, 3A) and, to a lesser extent, the later school stages. In the latter school periods, the more difficult sub-tests (5, 5b, 8a, and 8b) and the easier and quicker ones (7a, 7b) maintain high discriminative ability between school phases (3B to 5B).
- The Top-RAN Battery’s unique features, notably its ability to maintain and accentuate performance differences between sub-tests even when only errors are considered and time variables are excluded, distinguish it from all other RAN tests and psychometric tests of optometric origin. This distinctiveness piques curiosity and underscores its potential for further exploration.

## 5 Discussion

### 5.1 Evolution of attentive, oculomotor, and executive reading skills

Numerous experimental paradigms have tested the link between saccades and executive functions (e.g., Antisaccade: Everling and Fischer, 1998; Ouerfelli-Ethier et al., 2018. Countermanding task: Stuphorn and Schall, 2006; Wong and Shelhamer, 2012. Memory Saccade: Loe et al., 2012. Predictive Saccade: Lukasova et al., 2016. For latency and duration of fixation effects see Fadardi et al., 2022). However, their link to reading ability is either interpreted as an issue co-occurrent with dyslexia (Biscaldi et al., 2000) or has been interpreted as a one-way causal link, meaning that the reading issues are causing the attentive, oculomotor, executive problems (Chamorro et al., 2017). The Top-RAN is primarily a RAN test with minimized PA components, introducing modular visuospatial difficulties through differential metrics, distractors, and crowding, creating conditions for the intervention of executive functions of error monitoring and management. Importantly, visuospatial challenges devised in the paradigms of subtests 3, 4, 5, 5b, 9, and 10 intended to test mainly the far right part of the span as the carrier of pure spatial localization processing. Unlike the other oculomotor experimental paradigms related to executive functions, those in the Top-RAN battery do not contain cues that appear peripherally (abrupt onset) and are essentially static. This feature (in addition to the proximity between performance in RAN tasks and reading skills that has been confirmed countless times) makes us assume deep ecological proximity between the proposed test battery and reading.

Our analyses allow us to claim that visuospatial reading skills that require error monitoring and management are implicitly developed as traditional reading skills develop. Our results support the hypothesis that developing these oculomotor, attentional, and executive skills depends primarily on school learning and growth. The subject of further investigation will be how much these visuospatial skills correlate with the passage or word reading skills or other skills generally acquired at school (e.g., passage writing, spatial arrangement of numbers in writing on the paper). If our results were confirmed, it would mean that *intelligent* oculomotor skills are involved in reading and connected mainly with attention and executive functions. This new perspective would give value to what Kennedy (2003) stated, “[…] reading should not be seen as *surrogate listening*.”

Beyond the relationship between the Top-RAN test and traditional reading, we believe these results are significant because they reveal the existence, implicit evolution, and automation of voluntary gaze (and attention) orientation skills—free from integration with gross motricity—which the subject can use in a wide variety of contexts and activities.

### 5.2 Error monitoring and correction

In the past, the RAN literature has minimized the influence of errors on the predictive relationship between RAN and reading. However, the psychometric tests of oculomotor skills did not leverage errors (except as execution time correctives) to classify the specific oculomotor difficulties of clinical cases. Moreover, neither of the two types of study considered self-corrections. The Top-RAN test—by explicitly affecting the possible attentional-oculomotor-executive difficulties through the increase of the challenges imposed by the task, distractors, and crowding—carries richer information: We show how mistakes and self-corrections can be utilized to classify subjects in subpopulations (clustering) and with respect to known features (e.g., grade).

If errors and self-corrections are essential in the evolution of performance, it would be necessary to study the different types of errors, their neuro-functional causes, and their development. Moreover, suppose that the verbal manifestation of errors is progressively canceled through learning. In that case, there could be a period of interregnum in which errors would not be absent but rather managed implicitly and gradually in more automated and efficient ways. The study of this optimization process would highlight the evolution of online error tracking and handling skills as essential executive functions. A longitudinal error monitoring/handling study could answer questions such as: How does error evidence accumulate? Is there a threshold beyond which the subject “must” self-correct? Is there a developmental trend in error-tracking skills? Is there a method to increase error monitoring and management skills in individuals with executive difficulties (ADHD; ADD; Asperger’s; Dyslexic; Dysgraphs; Dyspraxic)?

### 5.3 Future research directions: where-crowding and where-attention

On the topic of the relationship between saccadic computation and crowding in the past, there have been conflicting claims about the influence of oculomotor computation on crowded target discrimination (Harrison et al., 2013; Ağaoğlu et al., 2016). In visual search tasks, progressively increasing crowding increases search times by up to 76% more (Vlaskamp and Hooge, 2006). In reading, McDonald (2006) finds that for the same angular word size, the more letters contained in the word (the greater the inter-letter crowding), the greater the number of fixations, and these fixations will be longer. One study relevant to our work is that of Greenwood et al. (2017), conducted to understand whether variations in visual sensitivity across the visual field (anisotropy) inherent in visual acuity and crowding (identification) and saccadic eye movements (localization) depend on a single common source, a single brain map of space. Despite the remarkable individual differences among the various subjects who participated in the proposed experiment, the analyses conducted by the authors reveal dissociations that rule out the existence of a shared reference representation for identification and saccadic computational processes. The global saccade effect (e.g., Walker et al., 1997), which brings the saccade landing point to an intermediate position between targets and distractors, depends on the proximity between elements and ultimately on their grouping by proximity (e.g., Findlay et al., 1993; for an application to word targeting, see Vitu, 2011); whereas accuracy in perceptual target identification (conditioned by crowding) was found to be independent of saccadic errors. Notably, the paradigm in Experiment 1 required the performance of a dual task (crowded target discrimination + saccadic computation toward the target itself), and in their conclusions, Greenwood et al. state, “Our participants were clearly able to trade their precision between the two processes; saccadic precision was highest in trials where crowded identification was incorrect and vice versa.” This bargaining implies the intervention of executive functions capable of managing the balance between the resources allocated to the two tasks. Thus, the use of paradigms such as subtests 2, 6, 8a, 8b, and 10 that simultaneously test identification (through the naming of the crowded digit) and localization (through the use of horizontal, vertical, or two-dimensional groupings that solicit the global saccade effect) can also test the executive functions that distribute attentional resources across the two tasks.

In our analyses, one noteworthy finding concerns the progressive improvement in the subject’s ability to manage two-dimensional crowding during voluntary gross-motor-free orientation of attention and saccades. This result was particularly evident in terms of execution time, the number of errors, and the specificity of those errors (and self-corrections). The absence of significant differences between BGT members (namely, subtests 1, 2, and 6) in all grade levels rules out specific difficulties in dealing with one-dimensional crowding. Nevertheless, in subtests 10, 8a, and 8b, there is significant differentiation in execution time, errors, and self-corrections compared to other subtests and their precursors within the respective sequence (see Section 3.3). This distinction indicates the development of an ability to manage gaze shifting, particularly in environments characterized by two-dimensional crowding. It is impossible to attribute this considerable differentiation solely to the difference between pseudo-reading tasks and alternate-column or serpentine-path reading tasks. Furthermore, the highlighted errors and self-corrections affect spatial localization (see the specific errors and self-corrections for individual subtests listed in Table 4). Future research shall verify the existence and evolution of these spatial localization difficulties to determine whether a form of crowding that affects only the visuospatial aspects of attention orientation (*where-crowding*) exists.

Since the ability to resist distractors determined by antigrouping also appears to be subject to the same developmental trend that has occurred in crowding management skills (see Section 3.3), and taking into account what was discussed in the part of the Review section devoted to word length span regarding the extreme right-hand side of the perceptual span, one could hypothesize the existence of a form of the implicit or explicit orientation of attention not closely related to identification but devoted solely to localization. It would be a *where-centric where-attention* that precedes and guides *where-to-what attention* like a pathfinder capable of creating a stabilizing and consolidating visuospatial context of whole processing.

Finally, we would like to emphasize the possibility that in the future, our battery of tests may be used not only in the area of learning disabilities but also in the area of post-traumatic outcomes in sports medicine, as has already been the case for more than two decades in the King-Devick test (e.g., King et al., 2020).

## Data availability statement

The datasets presented in this study can be found in online repositories. The names of the repository/repositories and accession number(s) can be found at: https://github.com/LimenResearch/topran_stats/tree/main/data.

## Author contributions

ML: Conceptualization, Data curation, Formal analysis, Methodology, Project administration, Software, Supervision, Writing – original draft, Writing – review & editing. DM: Project administration, Supervision, Writing – review & editing. CM: Project administration, Resources, Writing – review & editing. MP: Project administration, Resources, Writing – review & editing. AM: Resources, Writing – review & editing. MB: Writing – original draft, Writing – review & editing.

## Ethics statement

Ethical approval was not required for the study involving human samples in accordance with the local legislation and institutional requirements. Written informed consent for participation in this study was provided by the participants’ legal guardians/next of kin.
