# Supplementary material for: Visuospatial, oculomotor, and executive reading skills evolve in elementary school, and errors are significant: a topological RAN study
Source: Front Psychol. 2024 Jun 6;15:1383969. doi: 10.3389/fpsyg.2024.1383969 (PMC11188999; doi:10.3389/fpsyg.2024.1383969)
Supplement: Supplementary file 1 [file Table_1.pdf]

Top-RAN Supplemental Materials

By Mario Lecce, Daniela Miazza, Carlo Muzio, Maria Parigi, Alessandra Miazza, Mattia G. Bergomi

1. Data

1.1 Structure

1B, 2A, 2B, 3A, 3B, 4A, 4B, 5B – Scholastic phases, where digit stays for grade (e.g. 1 = first grade) and letter stays for trimester (A = first trimester; B = third trimester).

CR – Carriage Return. Large saccade starting from the last or second-to-last digit of a row, toward the left, to reach the beginning of the next row. In tests 3, 4, 5, 5b, 11, 12, and 13, the targeting of the beginning digit of the row can be variously incorrect. In tests 4, 5, 5b, 12, and 13, CR may (fully or partially) correct previously committed LC errors.

LC – Lane Change. While proceeding to the right, the subject involuntarily deviates diagonally from the perfectly horizontal row he or she travels, moving to the next higher or lower row. There are also corrective (of previous errors) LCs: 1) retrograde LCs (returning to the second-to-last digit or third-to-last digit read), which then cause rereads; 2) lateral step LCs to the digit immediately above or below the last digit read, which do not cause rereads. 3) CR-Corrective LCs, toward the beginning digit of the row. At last, anomalous LCs: a) deviate two (or more then two) higher or lower rows, sometimes in the reverse direction; b) deviate to the next higher or lower row but skip a digit. c) sometimes have corrective intentions.

Off-path – In following the serpentine path of digits, the subject suddenly changes path, unable to locate the next digit. After this change, the subject may perform a particular section in the opposite direction from the normal one. This error often results in other off-paths intended to minimize the omitted digits.

1.2 General Information

Column	Description
Id	Unique number assigned to each student.
School	Name of the School Attended by the student.
Section	School Section.
Entry Year	First test administration (school year).
Sex	Sex of the student (male or female).
Non-native Speaker	True if Italian is not the native tongue of the student.
Age	Age at time of administration (years).
Scholastic Phase	School year and trimester at test administration.
Acquisition Period	Date of test administration (mm/yyyy)

1.3 Common Variables (all subtests)

Column	Description
Time	Total time per subtest (sec)
Average Time per Digit	Average time per digit (sec), subtracting omissions and adding re-readings.
Omissions	Number of omitted digits.
Additions	Number of re-readings.
Addition-Subtraction Ratio	Addition — Omissions.
Self-correction Label	Self-corrected errors in attributing the correct verbal label.
Self-correction Omissions	Number of omitted digits, subsequently read (regression) in a self-correction process.
Self-corrections	Total number of self-corrections.
Label Errors	Non self-corrected labelling errors.
Order Errors	Adjacent digits labelled in reversed order.
In Loco Re-readings	Number of re-readings of the same digit. It includes on-site, self-correction re-readings.
Secondary in Loco Re-readings	Self-correction re-readings of the same digit.
Non-secondary Post-regression Re-readings	Re-readings of one or more digits through the execution of a regression, without omissions, and labelling errors.
Secondary Post-regression Re-readings	Re-readings during regression to correct an error.
Sign Loss	Sign loss.
Slowdown	Hesitations due to localization problems and self-corrections
Global Movements	Head and torso movements or excessive leg movements.
Fatigue	Signs of fatigue.
Finger	Usage of a finger to locate the digits (blocked by the administrator).

1.4 Typical errors and self-corrections

1.4.1 Subtests 3, 4, 5, 5b, 11, 12, and 13

Column	Description
CR Errors	Carriage-return (CR) errors.
Bustrofedic CR	Carriage return performed through a long regression along the entire length of the line of digits just read, until student gets to the beginning of the line and performs a LC down, on the beginning of the next line.
Non-self-corrected Spatial CR	Number of non-self-corrected carriage return errors.
CR Self-corrections	Number of self-corrected carriage returns.
LC Errors	Lane change (LC) without the purpose of carriage returns.
LC Self-corrections	LC self-corrections.
Memory-Based CR	Carriage errors correcting ascending or descending LCs while reading the same rightward saccadic progression The patient uses the memory of the first digit of the line just read to locate the first digit of the next line.
Spatial CRs	Carriage return errors that: a) omit one or more rows of digits below the line just read; b) produce the re-reading of a line before the one just read; c) Produce the re-reading of the line just read.
Special LC	Anomalous LCs and carriage returns corrections
Ascending (Descending) Distal LC	Ascending (resp. descending) LC apparently not justified by perceptual criteria of proximity.
Ascending (Descending) Proximal LC	Ascending (resp. descending) LC apparently justified by perceptual criteria of proximity.
Second Transit Re-readings	Re-reading errors due re-reading of the same line just read or ascending LCs.
Non-self-corrected LC	Non-self-corrected LCs.

1.4.2 Subtest 5b

Column	Description
Spatial Memory Based CRs	Incorrect carriage returns at the last digit (bottom right).
Self-Corrected Channelled LC	Self-corrected channeled LCs.
Consecutive Channelled LC	Subsequent channeled LCs.
Isolated Channelled LC	Channeled LCs neither followed nor preceded by other channeled LCs.
Net Channelled LC	Non-self-corrected channeled LCs.
Channelled LC	Total LCs following the gray or white oblique stripes

Column	Description
Correction of channelled end-of-line LC	Channeled LC corrections performed at the end of the row.
Anomalous LC	Number of anomalous LCs.
Compensatory Anomalous LC	Number of abnormal LCs correcting for LC errors.
Non-Self-Corrected Ascending-Descending LC	Number of non-channeled non-self-corrected LCs.
Start of Line Omissions	Omissions of the first digit of each row.
First Channelled LC Index	Occurrence index of first channeled lane-change error.
First Non Self-corrected Channelled Lane Index	Occurrence index of first non self-corrected channeled LC.
First LC Index	Occurrence index of the first LC (any).
Direct (Reverse) Site of First Channelled LC	Digit coordinates—top to bottom, left to right (resp. bottom to top, right to left)—, of the first channeled LC.
Direct (Reverse) Site of First Not-Corrected Channelled LC	As previous row for the first non-self-corrected channeled LC occurs.
Direct (Reverse) Site of First LC any	As previous row, for any LC.
Absolute disorientation	The administrator intervenes to signal a new, correct starting point.

1.4.3 Subtests 7a, 7b, 8a and 8b

Column	Description
LC errors	During the progressive saccade from left to right, the student skips down or up one or more lines.
LC self-corrections	LC self-corrections.
Column Self-Corrections	Self-correction of column errors.
Not-Self-Corrected Column error	Non-self-corrected column errors.
Carriage return errors	Carriage return errors.
Sum of Column Errors	Total column errors.
Left Column errors	Digit localization errors on the left-hand column.
LC self-corrections	LC self-corrections.
LC errors	LC errors.
Absolute disorientation	Situations of absolute disorientation. The administrator intervenes to signal a new, correct starting point.

1.4.4 Subtest 9 and 10

Column	Description
Random Off-path	The student reads a digit out of the assigned path and far from the target digit.
Pseudo-Reading Off-path	Off-path reading performing a long oblique saccade downwards (return carriage simulation).
Perceptual Off-path	Off-path reading of a digit spatially close to the target digit.
Total Off-paths	Sum of all out-of-path errors.

2 Data Analysis

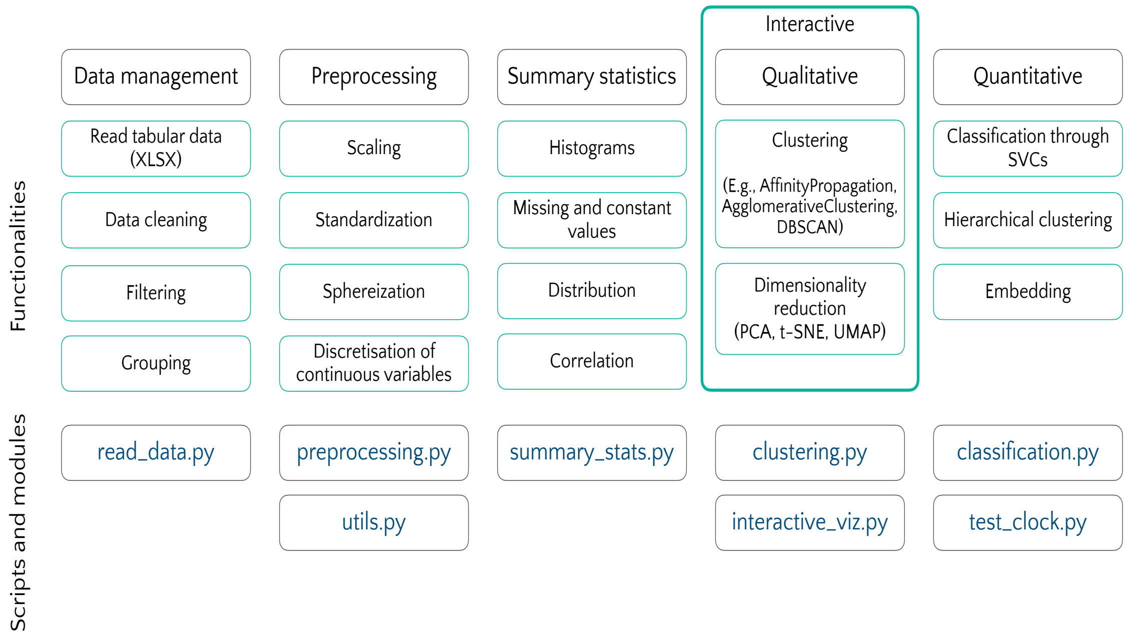

Figure 1: Top-RANStats.py structure. The main functionalities of the package allow for swift data cleaning, analysis and visualization of tabular datasets.

2.1 Summary statistics

Age
Real number ($\mathbb{R}_{\geq 0}$)

HIGH CORRELATION (This variable has a high correlation with 2 fields: Scholastic Phase, Acquisition period)

**Distinct**	441	**Minimum**	6.237899543
**Distinct (%)**	83.8%	**Maximum**	11.34155251
**Missing**	0	**Zeros**	0
**Missing (%)**	0.0%	**Zeros (%)**	0.0%
**Infinite**	0	**Negative**	0
**Infinite (%)**	0.0%	**Negative (%)**	0.0%
**Mean**	8.018378995	**Memory size**	4.2 KiB

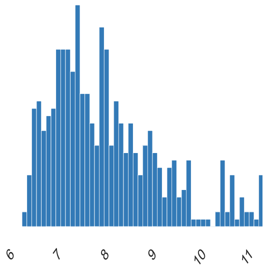

Quantile statistics

**Minimum**	6.237899543
**5-th percentile**	6.579452055
**Q1**	7.149086758
**median**	7.82260274
**Q3**	8.672545662
**95-th percentile**	10.50410959
**Maximum**	11.34155251
**Range**	5.103652968
**Interquartile range (IQR)**	1.523458904

Descriptive statistics

**Standard deviation**	1.15621531
**Coefficient of variation (CV)**	0.1441956423
**Kurtosis**	0.3419464207
**Mean**	8.018378995
**Median Absolute Deviation (MAD)**	0.7283105023
**Skewness**	0.9257139628
**Sum**	4217.667352
**Variance**	1.336833842
**Monotonicity**	Not monotonic

Figure 2: Summary statistics for the Age column obtained through the Pandas profiling package.

We produce summary-statistics reports via Pandas Profiling available at https://github.com/ydataai/ydata-profiling. We apply the main routine offered by the package to the dataframe encompassing the entire dataset (see 1) computing minimal statistics, namely histograms and distributions of discrete and continuous variables, respectively, counting missing entries, correlation across continuous variables, and enumerating repeated rows. We generate a more detailed Pandas-profiling report for the dataframes associated with each subtest.

2.2 Dimensionality reduction

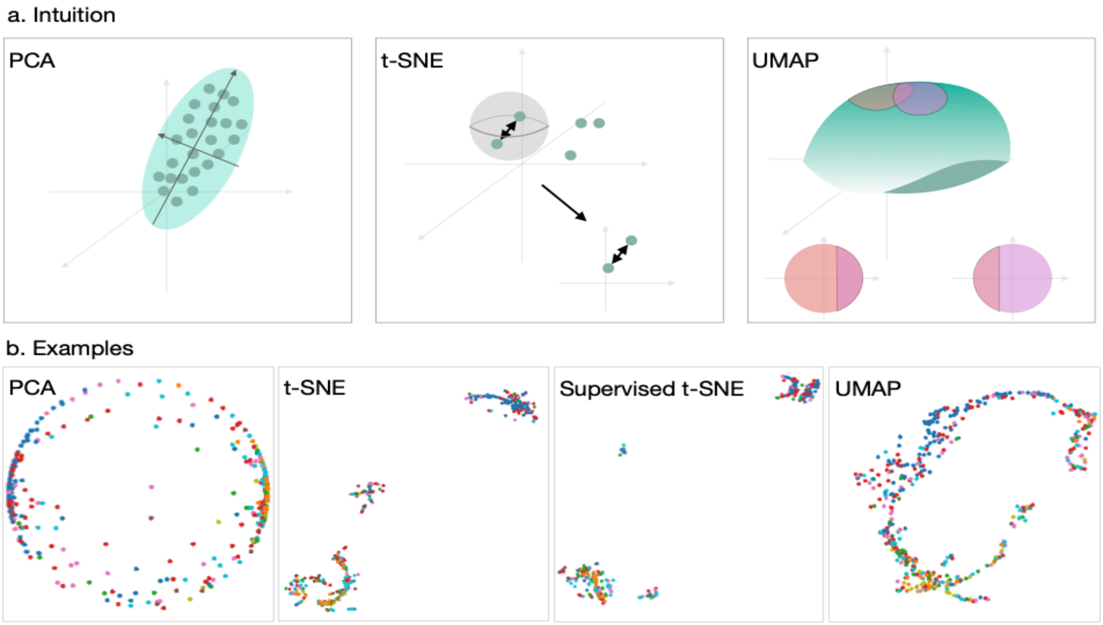

Figure 3: Dimensionality reduction. a. Intuitive depiction of the dimensionality reduction algorithms leveraged by the Top-RANStats package. While PCA retrieves orthogonal axes of maximum varianve, t-SNE and UMAP try to preserve local distance between points. b. Interactive visualization obtained via Tensorboard (Abadi et al., 2016).

High-dimensional data can be explored by finding optimal, low-dimensional projections in an unsupervised fashion, or constraining on a given labelling function. In Top-RANStats, dimensionality reduction algorithms are used for preprocessing data, or produce interactive visualizations. We leverage the following algorithms:

- Principal Component Analysis (PCA),
- t-Distributed Stochastic Neighbor Embedding (t-SNE))VanderMaatenand and Hinton (2008);
- Uniform Manifold Approximation and Projection (UMAP) McInnes et al. (2018).

See fig. 4 for intuition and examples.

2.3 Clustering

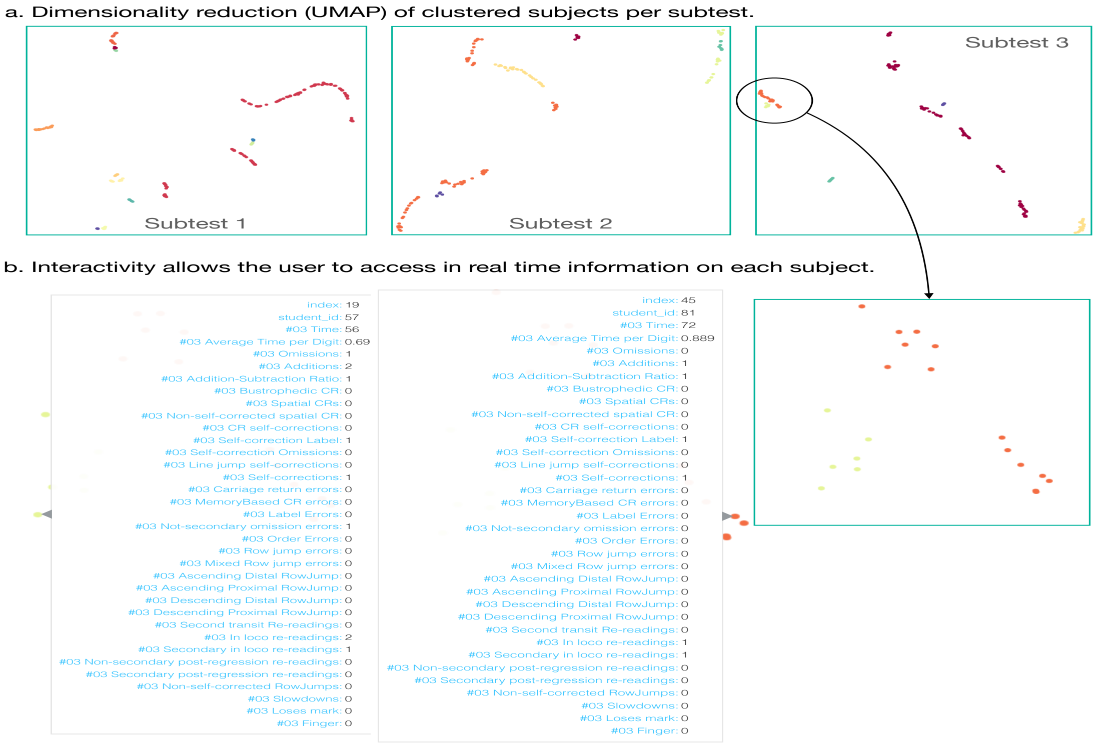

Figure 4: Clustering. a. Cluster obtained by considering students' performance per subtest. Noisy points are discarded. Two-dimensional projections of high-dimensional clustered points are obtained through UMAP (see fig. 4 and McInnes et al., 2018). b. Mouse hover allows the user to visualize performance, and general information of the student associated with each point.

The *Top-RANCluster* class allows to swiftly apply clustering algorithms to each subtest dataset. The available algorithms are

- AffinityPropagation,
- AgglomerativeClustering,
- Birch,
- BisectingKMeans,
- DBSCAN,
- FeatureAgglomeration,

- KMeans,
- MeanShift,
- MiniBatchKMeans,
- OPTICS,
- SpectralBiclustering,
- SpectralClustering,
- SpectralCoclustering,

as implemented in the *scikit-learn* package (Pedregosa et al., 2011). The user can choose one of the algorithm listed above, parametrize it via a dictionary, and provide an input dataframe, specifying the names of feature and label columns. The class generates interactive plots as showcased in fig. 4. High-dimensional points are projected to a two-dimensional space via UMAP (McInnes et al., 2018) and color coded according to their cluster labels. Noisy points are excluded from the visualization.

2.4 Classification

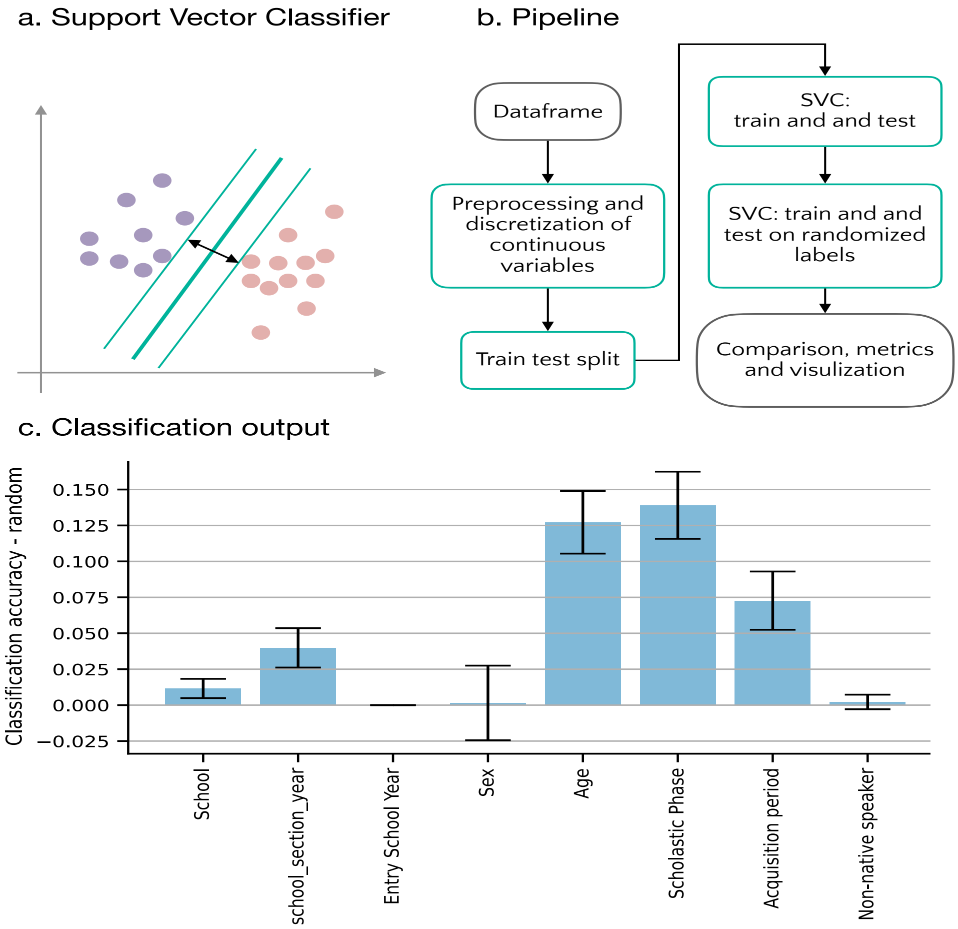

Figure 5: Classification pipeline. a) Support Vector Machines aim to find a hyperplane that optimally separates samples belonging to different classes, i.e., the optimal hyperplane is the one realizing the maximum distance between the two classes. b) Analysis pipeline, from preprocessing to random labelling test. Data are organized in features and targets and split into training and test set. After fitting and testing the Support Vector Classifier, we iterate the procedure on random permutations of the target labels. This procedure allows us to produce the barplot in panel c.

We leverage Support Vector Machines (SVMs) to discover potential subtest biases. The *classify.py* script encompasses the functions necessary to instantiate, parameterize, fit, and test a Support Vector Classifier. The same script provides specific methods to discretize continuous variables, and visualize the results obtained by computing the test accuracy of the trained model, or the distribution between the model's accuracy on test, against the accuracy obtained training the models on randomly labelled data for n iterations. Figure 5 gives an intuition on SVMs, provides the analysis pipeline adopted in the manuscript, and an example of visualization of the performance of the model.

2.5 Subtest Metrics and Hierarchical Clustering

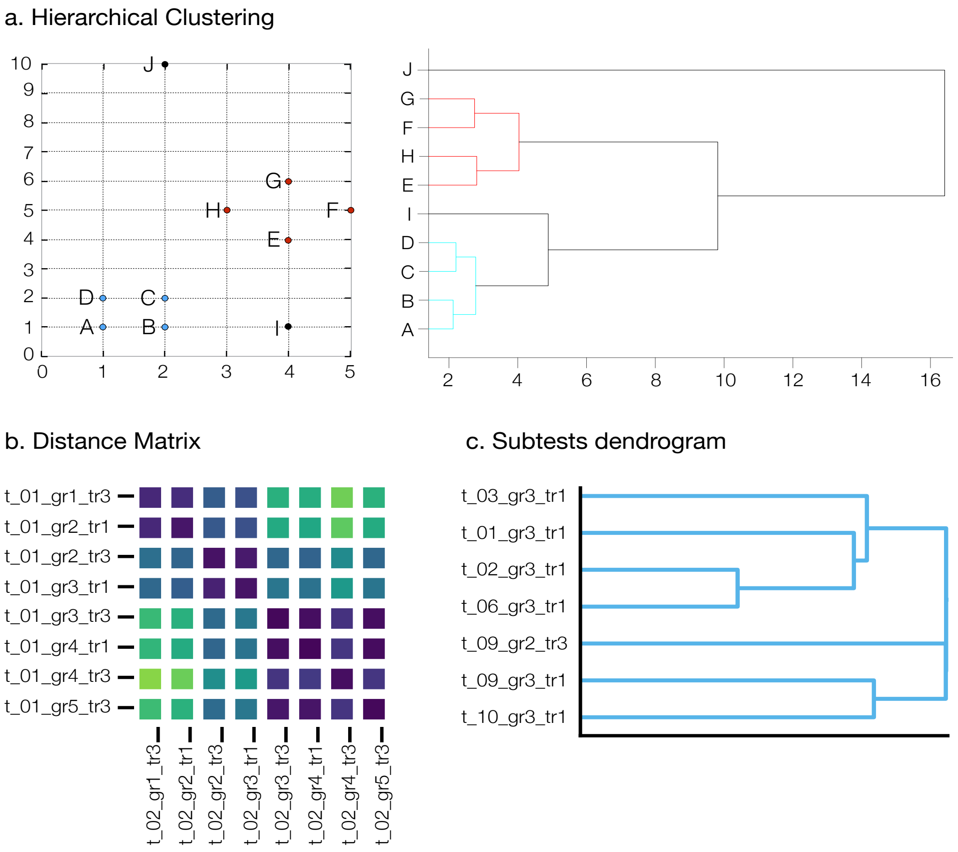

Figure 6: Distance between subtests and hierarchical clustering. a. Pairwise distances computed between points of a two-dimensional point cloud are depicted as a dendrogram. Observe how clustered points in the plane are represented by low-distance branches in the dendrogram, whilst outliers merge only at high distance. b. Distance matrix obtained comparing juxtaposed feature vectors of the students' performance in subtests 1 and 2, grouped by scholastic phase. Labels are organized as subtest number, grade, and trimester. c. A sample of the dendrogram obtained considering the same juxtaposed feature vectors across all subtests.

Summary statistics, namely count, mean, standard deviation, minimum, percentiles (25, 50, 75), and maximum, serve as fingerprint of students' performance in each subtest. We utilize this representation to measure distance across subtest and by grouping students with respect to any relevant information. In symbols, let $S^i = \cup_j G_{i,j}$ be the collection of grouped user performance for the i-th subtest, and $\sigma: U_i \to \mathbb{R}^8$ the function mapping each U_i to the statistical measures mentioned above. With a notation abuse, let $\sigma(S_i) = \{\sigma(G_{i,j})$ for every $G_{i,j} \subset S^i\}$, and analogously $\sigma(S)$ the collection of the summary statistics computed for all subtests and subgroups. A natural metric between subtests is the Euclidean distance measured between juxtaposition of the vectors in each S_i. However, as detailed in 1, not all subtests share the same types of error

descriptors. Thus, we select statistics associated with common errors across subtests $\hat{S}_i$, and juxtapose those vectors, obtaining, for each subtest, an element $\hat{s}_i \in \mathbb{R}^{80}$.

We can now measure Euclidean distance between the 80-dimensional vectors computed via the procedure described above. That distance allows us to cluster students' performance by subtest and specific features hierarchically. We represent these clusterings as dendrograms. See fig. 6.

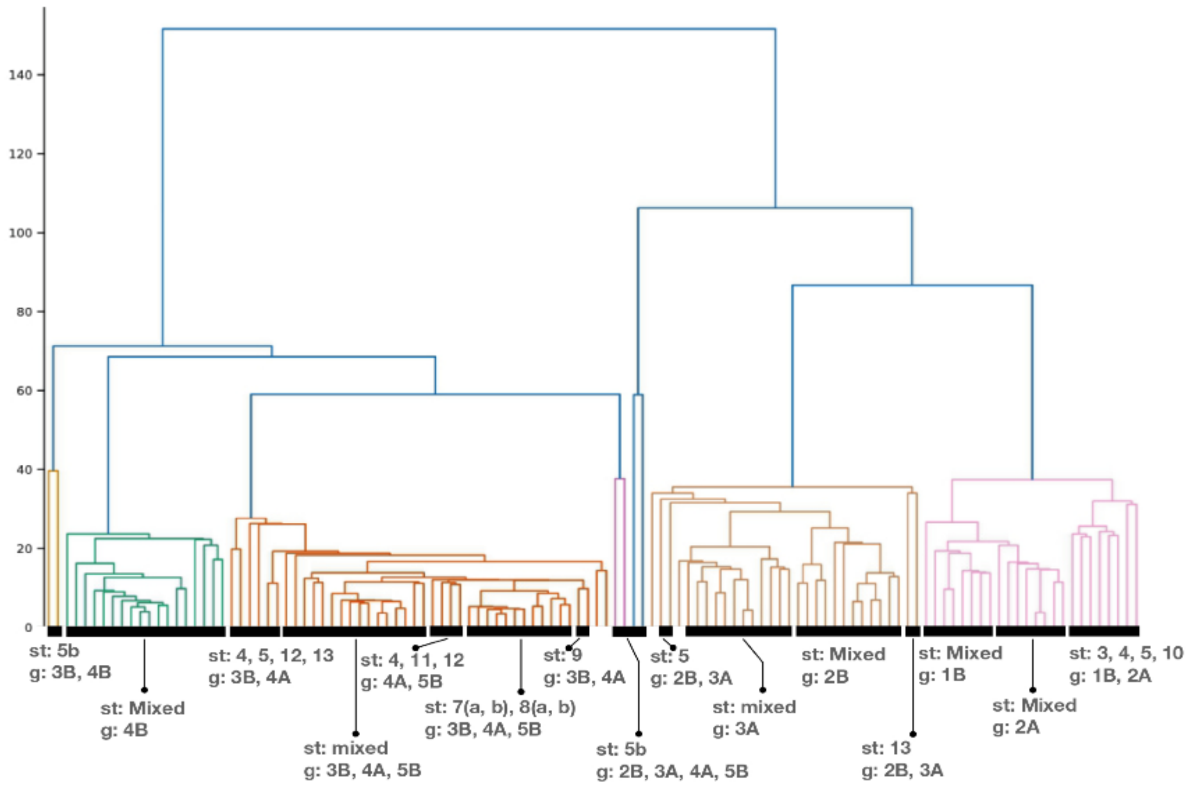

Figure 7: No-Time Dendrogram

3. Subtest Description

Test	Task	Distractor	Distractor Arrangement	Item n and series	Introduction Period	Complexity Determinant	Facilitating Factors	Max Distance items	min Distance items
#1 Uncrowded Rows	Pseudo-Reading	—	—	80 - ABCD	1B	—	PPB	line spacing 132 px	3 character spaces
#2 Crowded Rows	Pseudo-Reading	Horizontal Crowding	Reduced Inter-Target distance	80 - CDAB	1B	Horizontal Crowding	PPB	Line spacing 132 px	0 character spaces - 58 approximation
#3 Reduced Antigrouping	Pseudo-Reading	Vertical Antigrouping canceled by horizontal stripes	—	80 - CDAB	1B	Target by target H Oculomotor Computation	Alternating Gray & White Horizontal Stripes<br>PPB	8 character spaces	Line spacing 14 px
#4 Vertical Antigrouping	Pseudo-Reading	Vertical Antigrouping	Grouping by serpentine columns	80 - CDAB	1B	#3 + CR computation + handling of horizontal saccades vertical error	Vertical Subitizing<br>Verbal WM<br>PPB	8 character spaces	Line spacing 12 px
#5 Colored Vertical Antigrouping	Pseudo-Reading	Vertical Antigrouping reinforced with areas and colors	Grouping by serpentine columns	80 - CDAB	1B	#4 + Grouping salience by area and color	Vertical Subitizing<br>Verbal WM<br>PPB	8 character spaces	Line spacing 12 px
#5b Dual Antigrouping	Psudo-Reading	#4 + Oblique Antigrouping	#4 + Grouping by oblique stripes	80 - CDAB	2B	#4 reinforced handling of horizontal saccades vertical error	Vertical Subitizing<br>Verbal WM<br>PPB	8 character spaces	Line spacing 12 px
#6 Vertically Crowded Columns	Vertical Reading	Vertical Crowding	Reduced inter-target Distance	80 - BADC	1B	Vertical Crowding	PPB	Inter-column > 16 character spaces	Line spacing 11 px
#7a Uncrowded Voluntary Saccades	Reading by jumping between alternate columns	—	—	20 - A	1B	—	PPB	Inter-column space > 7 character spaces	Line spacing 14
#7b Color Uncrowded Voluntary Saccades	Reading by jumping between alternate columns	Two-dimensional Attenuated Crowding	columns of black digits surround the red target-columns (3rd and 3rd-last columns)	20 - B	1B	Two-dimensional crowding reduced by target-column salience	PPB<br>Red coloring of target columns	Line spacing 14 px	Inter-column 1 character space -97 Approximation
#8a Crowded Voluntary Saccades	Reading by jumping between alternate columns	Two-Dimensional Crowding	Columns of black digits surround the 3rd and 3rd-last black target-columns	20 - C	1B	Two-Dimensional Crowding	Bidimensional Subitizing<br>Verbal WM	Line spacing 14 px	Inter-column 1 character space -97 Approximation
#8b Crowded Voluntary Saccades	Reading by jumping between	Two-Dimensional Crowding	Columns of black digits surround the 3rd and 3rd-last black target-columns	20 - D	1B	Two-Dimensional Crowding	Bidimensional Subitizing<br>Verbal WM	Line spacing 14 px	Inter-column 1 character space -97 Approximation

Test	Task	Distractor	Distractor Arrangement	Item n and series	Introduction Period	Complexity Determinant	Facilitating Factors	Max Distance items	min Distance items
	alternate columns								
#9 Switch online	Serpentine Path	—	—	88 - AB'CD	1B	Target by target Oculomotor / span flexibility	PPB	4 character spaces	Line spacing 12 px
#10 Switch in Crowd	Serpentine Path	Two-Dimensional Crowding	Pseudo-Digits surrounding the serpentine path	88 - AB'CD	1B	#9 + two-dimensional crowding	PPB	4 character spaces	Line spacing 12 px
#11 Reverse Reading	Reverse Reading (derived from #3)	Vertical antigrouping canceled by horizontal stripes	—	80 - CDAB	2B	Target by target Reverse Oculomotor computation	Vertical Subitizing<br>Verbal WM<br>PPB	8 character spaces	Line spacing 14 px
#12 Antigrouping Reverse Reading	Reverse Reading (derived from #4)	Vertical Antigrouping	Grouping by serpentine columns	80 - CDAB	2B	#11 + Reverse CR + handling of horizontal saccades vertical error	Vertical Subitizing<br>Verbal WM<br>PPB	8 character spaces	Line spacing 12 px
#13 Colored Antigrouping Reverse Reading	Reverse Reading (derived from #5)	Vertical AntiGrouping reinforced with areas and colors	Grouping by serpentine columns	80 - CDAB	2B	#12 + Grouping salience by area and color	Vertical Subitizing<br>Verbal WM<br>PPB	8 character spaces	Line spacing 12 px

CR = Carriage Return

PPB = Parafoveal Preview Benefit

WM = Working Memory

4. Word Targeting and Spatial Indexing in Reading

In this section, we will give an overview of what is known about the use of spatial qualities of text for the purpose of oculomotor computation and correct spatial localization of words. We will refer, in particular, to the mechanisms of word targeting along the line of text, return sweep saccading, and executing of long- and short-range voluntary regressions. In this context, we will introduce the concept of spatial indices and their use during reading in determining oculomotor inhibition of return (O-IoR).

4.1 Word Targeting

Word targeting within a text line is a crucial aspect of oculomotor computation. In word targeting research, two parameters are relevant: *where* the word is fixated (saccade metrics usually expressed in character space, from launch point to landing point) and *when* the eyes will move (duration of fixation). For example, the probability of refixation of a word is affected by the location of the saccade landing on it (McConkie et al., 1988; 1989). Children and adults aim their saccades toward the center of the word (Vitu et al., 2001), and the farther away from the center of the word the saccade lands, the greater the likelihood of refixing the same word (Joseph et al., 2009). Blythe and Joseph (2011) argue that off-center initial fixation on the word requires refixation: extreme viewing positions would not allow "extraction of the visual information necessary for full lexical identification of the word." Thus, the word centers constitute an actual Optimal Observation Position (OVP), and this position can be targeted through the perception of word length determined by inter-word spaces (e.g., Pollatsek et al., 2008).

4.2 Return-sweep saccades

Recent research has also dealt with return-sweep saccades, their metrics, and development. These saccades are intended to carry the gaze from the end of one line to the beginning of the following line. The return-sweep saccade, as a markedly longer saccade than the intra-row saccades, is more affected by possible errors of muscular origin (McConkie et al., 1988). Therefore, the adult often produces hypometric saccades that require a

subsequent corrective saccade (Heller, 1982; Hofmeister et al., 1999; Parker et al., 2017; Rayner, 1998). The localization of the landing at the beginning of the following line does not appear to be affected by word length, as is the case for intra-line word targeting (Radach & Heller, 1993), probably because the subject cannot make use of parafoveal or peripheral information about word length as efficiently as is feasible during progressive saccades. In their study comparing the performance of 47 adults and 48 children, Parker et al. (2019) find that the launch point of the carriage saccade in children is closer to the end of the line and targets a position closer to the left margin of the text, compared to adults. In addition, children are more likely to perform a corrective saccade after a carriage return.

Parker and collaborators also study the brief fixations that follow undershooting carriage returns, which they call "undersweep fixations" (Parker et al., 2017, p. 3), after which a corrective saccade always occurs toward the beginning of a line word. According to Becker (1976), each fixation followed by a corrective saccade is terminated early based on the spatial distance to the intended target: the greater this distance, the greater the likelihood of shortening the fixation and performing a corrective saccade. Slattery & Parker (2019) found that, despite the extreme brevity of these fixations in adults, they allow them to acquire information about the fixated word and the line start word toward which the corrective saccade is performed. These effects are also present in children aged 7 to 8 years (Parker et al., 2020).

4.3 Regressions

Despite what was said earlier about the visuospatial aspects of EMsR (Eye Movements in Reading), "spatial indices" are more rarely mentioned in this area of research than, for example, in the area of attention. For example, in research on Visual Search, spatial indices are understood as markers that facilitate responding and attract attention according to the task and voluntary aspects of the deployment of visual attention (Yantis & Johnson, 1990; Yantis & Jonides, 1990; 1991). Another example is research using the MOT paradigm (Multiple Object Tracking; Pylyshin & Storm, 1988), which goes as far as delineating a maximum number of 4-5 spatial indices deployable simultaneously on moving objects.

In reading research, spatial indices have been studied concerning regressions in two mnemonic meanings: one concerning previously fixated words far from the current fixation and one concerning words close to the currently fixated word (precisely the n-1 word).

Already, Kolers (1968) noted that inter-word regressions do not hinder the process of global meaning extraction. Later, many authors, because of the discovery of greater accuracy in saccadic programming of the return toward words immediately preceding fixation (Radach & McConkie, 1998), proposed visuospatial-based word indexing appropriate for selective programming of regressions (Zechmeister et al., 1975; Kennedy, 1987, 1992, 2000; Kennedy et al., 2003; Kennedy & Murray, 1986). These spatiotopic indices inform the syntactic processor that word *N-1* does not follow word n fixed; therefore, they would help to fixate word *N + 1* directly (skipping word *N*) following the fixation of word *N-1*.

Regarding long-range regressions, some studies have shown that readers tend to move their eyes to a region of the previously read sentence that is critical for disambiguating an ambiguous sentence encountered later (Carpenter & Daneman, 1981; Ehrlich & Rayner, 1983; Frazier & Rayner, 1982; Rayner & Frazier, 1987). However, Rawson and Miyake (2002) found that word localization skills in previously read text correlated more with readers' linguistic ability than their visuospatial abilities. Current researchers seem to agree that as long as the word is at a maximum distance of 10 character spaces from the fixated word n, the presence of a "labile" spatial index is plausible because of the extreme precision of the oculomotor calculation of regression, which can bring fixation very close to the center of the word (Hogaboam, 1983; Radach & McConkie, 1998; Vitu & McConkie, 2000). Nevertheless, beyond this distance, the reader is more likely to use verbal memory to reconstruct the location of the searched word. In their four experiments, InInhoff and Weger (2005) wanted to test the idea of the absence of actual global spatial indexing of text. They found that readers performed two steps to reach distant words: one that brought their eyes roughly close to the target words and a second that brought their eyes precisely and automatically to them. They conclude that, while precise spatial memory may not exist for longer than 50 ms (Werner & Diedrichsen, 2002), long-range regressions can make use of a form of "coarser

encoded spatial knowledge" (Inhoff & Weger, 2005, p. 17) that can be used to perform the first saccade that brings the eyes to an area close to the target word: then, through the use of verbal memory, precise localization can occur (see also Cane et al., 2012).

4.4 Oculomotor Inhibition of Return

The effect of oculomotor inhibition of return (O-IoR) manifests itself as an increase in fixation duration that precedes a regression (or progression) toward an already fixated word (Rayner et al., 2003; Weger & Inhoff, 2005; Henderson & Luke, 2012; for general framing of O-IoR, see Klein & Hilchey, 2011). In particular, Henderson and Luke's (2012) study determines the existence of an exact spatial index placed on the particular character previously fixated within the word being returned to fixate: the closer the return saccade was to that precise character, the greater the time spent on the fixation preceding it. This effect is present in both reading and pseudo-reading tasks. According to Henderson and Luke, this indicates that it is not a consequence of language processing but of oculomotor control.

Even after a long return-sweep saccade, the undersweep fixations studied by Slattery & Parker (2019) and Parker et al. (2020) can produce an O-IoR effect in both adults and children: this always manifests itself as a temporal penalty associated with the new deployment of attention, when, after the corrective saccade toward the beginning of the line, the subject returns fixation toward the location of the undersweep fixation.

5. Explainability – Individual subtests' observations

- Subtest 5b is an outlier and requires a separate analysis. In this regard, the separation between the period before 3B onwards and the period from 2B to 3A is evident.
- Clusters 8a and 8b and clusters 7a and 7b are separated from other subtests' clusters and divided by period; this indicates that these paradigms are highly differentiated from the others.
- A group of basic subtests, 1, 2, and 6, is identified, which shows fewer differences between each other in all school periods. This minimal Basic Tests Group (BTG) corresponds to the One-dimensional Crowding Group. However, the BTG will tend to increase the number of participating subtests as the school progresses until it reaches its maximum number of participants at the end of fifth grade. This marked trend, in our opinion, confirms the presence of a developmental trend.
- Subtest 5 in first grade is an outlier, probably because it is challenging for age. The difference with the other subtests significantly diminished from 2A (it groups with the other most demanding subtests of the period: 4, 3, and 10) and then reduces slowly and quite progressively; however, it becomes part of the BTG only in 5B.
- Subtest 3 from 1B to 2B is one of the most challenging subtests: from 3A onwards, it falls into the BTG.
- Subtest 4, from 1B to 2A, is one of the most challenging subtests: from 2B to 3A, it falls into the BTG; it returns to differentiate from 3B to 4A; and finally, it falls into BTG from 4B onwards.
- Subtest 9 differs from basic tests only in 1B; it remains firmly among the BTG.
- Subtest 10 has a similar trend to 4: from 1B to 2A is one of the most challenging subtests: from 2B to 3A, it falls into the BTG; it returns to differentiate from 3B to 4A; and finally, it falls into the BTG from 4B onwards.
- Subtest 11 at its first administration (in 2B) is part of the BTG, with one exception in the 3B, where it differs by grouping abnormally with 4B subtests.

- Subtest 12, at its first administration (in 2B) differs markedly from the other subtests; in 3A, it is part of the BTG; in 3B, it returns to differentiate quite clearly; it will be part of the BTG only in 5B.
- Subtest 13 remains from the beginning (in 2B) and is differentiated from all other subtests: it will fall among the BTG only in 5B.

6. Explainability - Sequences Dynamics

6.1 AntiGrouping (subtests 3-4-5) sequence

6.1.1 All-inclusive Dendrogram. *Difference 3-4* – in the first two school ranges (1B / 2A and 2B / 3A), the differentiation between subtests 3 and 4 is stark; it persists in 3B and 4A; it is reduced but not canceled in 4B and 5B. – *Difference 4-5* – in 1B is extreme (5 outliers); high in 2A and 2B; first approach in 3A and difference maintained in 3B and 4A; further reduction in 5B.

6.1.2 NoTime Dendrogram. *Difference 3-4-5* – in 1B / 2A, the 3-4-5 subtests are very different outliers; the substantial difference remains in 2B (with subtests 3 and 5 outliers); in 3A the 3 and 4 get closer but the 5 remains an outlier; in 3B the difference between 3,4 and 5 reduces further and remains so in 4A; in 4B the differentiation between the three subtests is increased; in 5B the 3 and 4 subtests come closer, but the difference with 5 remains high.

6.2 Alternate columns in a two-dimensional crowding sequence (subtests 7a-7b-8a-8b)

6.2.1 All-inclusive Dendrogram. In the first two school stages (1B / 2A and 2B / 3A), the differentiation between subtests 8a and 8b and their precursors 7a and 7b is very sharp. This difference gradually fades in the later school stages until it almost disappears by the end of 5th grade.

6.2.2 NoTime Dendrogram. In 1B, the four subtests are all different from each other but grouped together; from 2A up to 3A, the 7a and 7b subtests differ from 8a and 8b; this difference is maintained in 5B where 7b and 8a come closer together.

6.3 Serpentine paths in two-dimensional crowding sequence (subtests 9-10)

6.3.1 All-inclusive Dendrogram. Differentiation between subtests 9 and 10 remains clear up to 2B; declines markedly in 3A; rises again in 3B and 4A; recedes in 4B and recedes again but to a lesser degree in 5B.

6.3.2 NoTime Dendrogram. In the 1B – 2A range, subtests 9 and 10 are very different since the subtest is one of the outliers (along with subtests 3-4-5). The difference narrows slightly in 2B and even more so in 3A; it remains on the level in 3B and 4A; in 4B, they are very close as well as in 5B.

6.4 Antigrouping, reverse read sequence (subtests 11-12-13)

6.4.1 All-inclusive Dendrogram. *Difference 11-12* – Highest difference in 2B; attenuates in 3A; in 3B, 4A increases; in 4B remains high; in 5B decreases. *Difference 12-13* – high in 2B; decreases in 3A; remains high in 3B, 4A, and 4B. It decreases but does not cancel in 5B.

6.4.2 NoTime Dendrogram. In 2B, subtests 11 and 12 are distinctly different, but even more so is 13, which is an outlier; in 3A, the difference between 11 and 12 is reduced, but 13 remains an outlier; in 3B, the three subtests differ more; in 4A and 4B the level of differentiation remains high; in 5B 11 and 12 come even closer, but the separation from 13 remains clear.

References

Abadi, M., Agarwal, A., Barham, P., Brevdo, E., Chen, Z., Citro, C., Corrado, G.S., Davis, A., Dean, J., Devin, M., et al., 2016. Tensorflow: Large-scale machine learning on heterogeneous distributed systems. *arXiv preprint*, arXiv:1603.04467. https://doi.org/10.48550/arXiv.1603.04467.

Becker, W. (1976). Do correction saccades depend exclusively on retinal feedback? A note on the possible role of non-retinal feedback. *Vision Research, 16*(4), 425–427. https://doi.org/10.1016/0042-6989(76)90209-1

Blythe, H. S. S. L., H. I. ,. Joseph. (2011). Children's eye movements during reading. In S. Liversedge S. P. ,. Gilchrist, I. D. ,. Everling (Ed.), *The Oxford Handbook of Eye Movements* (pp. 643–662). Oxford University Press.

Cane, J. E., Cauchard, F., & Weger, U. W. (2012). The time-course of recovery from interruption during reading: Eye movement evidence for the role of interruption lag and spatial memory. *The Quarterly Journal of Experimental Psychology, 65*(7), 1397–1413. https://doi.org/10.1080/17470218.2012.656666

Carpenter, P. A., & Daneman, M. (1981). Lexical retrieval and error recovery in reading: A model based on eye fixations. *Journal of Verbal Learning and Verbal Behavior, 20*(2), 137–160. https://doi.org/10.1016/s0022-5371(81)90357-1

Ehrlich, K., & Rayner, K. (1983). Pronoun assignment and semantic integration during reading: Eye movements and immediacy of processing. *Journal of Verbal Learning and Verbal Behavior, 22*(1), 75–87. https://doi.org/10.1016/s0022-5371(83)80007-3

Frazier, L., & Rayner, K. (1982). Making and correcting errors during sentence comprehension: Eye movements in the analysis of structurally ambiguous sentences. *Cognitive Psychology, 14*(2), 178–210. https://doi.org/10.1016/0010-0285(82)90008-1

Heller, D. (1982). Eye movements in reading. In P. Groner R., Fraisse (Ed.), *Cognition and eye movements* (pp. 487–498). Deutscher Verlag der Wissenschaften.

Henderson, J. M., & Luke, S. G. (2012). Oculomotor inhibition of return in normal and mindless reading. *Psychonomic Bulletin & Review, 19*(6), 1101–1107. https://doi.org/10.3758/s13423-012-0274-2

Hofmeister, R., J. ,. Heller, D. ,. &. Radach. (1999). The return sweep in reading. In T. Becker W. ,. Deubel, H. ,. Mergner (Ed.), *Current oculomotor research* (pp. 349–357). Springer.

Hogaboam, T. W. (1983). Reading patterns in eye movement data. In K. Rayner (Ed.), *Eye movements in reading: Perceptual and language processes* (pp. 309–332). Academic Press.

Inhoff, A. W., & Weger, U. W. (2005). Memory for word location during reading: Eye movements to previously read words are spatially selective but not precise. *Memory & Cognition, 33*(3), 447–461. https://doi.org/10.3758/bf03193062

Joseph, H. S. S. L., Liversedge, S. P., Blythe, H. I., White, S. J., & Rayner, K. (2009). Word length and landing position effects during reading in children and adults. *Vision Research, 49*(16), 2078–2086. https://doi.org/10.1016/j.visres.2009.05.015

Kennedy, A. (1987). Eye movements, reading skill and the spatial code. In A. M. Beech J. R. ,. Colley (Ed.), *Cognitive approaches to reading* (pp. 169–186). Wiley.

Kennedy, A. (1992). The spatial coding hypothesis. In K. Rayner (Ed.), E*ye movements and visual cognition: Scene perception and reading* (pp. 379–396). Springer-Verlag.

Kennedy, A. (2000). Attention allocation in reading: Sequential or parallel? In J. Kennedy A. ,. Radach, R. ,. Heller, D. ,. Pynte (Ed.), *Reading as a visual process* (pp. 193–220). Elsevier.

Kennedy, A., & Murray, W. S. (1986). Spatial Coordinates and Reading: Comments on Monk (1985). *Quarterly Journal of Experimental Psychology, 39*(4), 649–656. https://doi.org/10.1080/14640748708401807

Kennedy, C., A. ,. Brooks, R. ,. Flynn, L. A. ,. Prophet. (2003). The reader's spatial code. In H. Hyönä J. ,. Radach, R. ,. Deubel (Ed.), *The mind's eye: Cognitive and applied aspects of eye movement research* (pp. 193–212). North-Holland.

Klein, M. D., R. M. ,. Hilchey. (2011). Oculomotor inhibition of retum. In S. Liversedge S. ,. Gilchrist, L. D. ,. Everling (Ed.), *The Oxford Handbook of Eye Movements* (pp. 471–492). Oxford University Press.

Kolers, P. A. (1968). Foreword. In E. B. Huey (Ed.), The psychology and pedagogy of reading (pp. xviii–xix). MIT Press.

McConkie, G. W., Kerr, P. W., Reddix, M. D., & Zola, D. (1988). Eye movement control during reading: I. The location of initial eye fixations on words. *Vision Research, 28*(10), 1107–1118. https://doi.org/10.1016/0042-6989(88)90137-x

McConkie, G. W., Kerr, P. W., Reddix, M. D., Zola, D., & Jacobs, A. M. (1989). Eye movement control during reading: II. Frequency of refixating a word. *Perception & Psychophysics, 46*(3), 245–253. https://doi.org/10.3758/bf03208086

McInnes, L., Healy, J., Melville, J., 2018. Umap: Uniform manifold approximation and projection for dimension reduction. arXiv preprint arXiv:1802.03426. https://doi.org/10.48550/arXiv.1802.03426.

Parker, A. J., Kirkby, J. A., & Slattery, T. J. (2017). Predictability effects during reading in the absence of parafoveal preview. *Journal of Cognitive Psychology, 29*(8), 902–911. https://doi.org/10.1080/20445911.2017.1340303

Parker, A. J., Slattery, T. J., & Kirkby, J. A. (2019). Return-sweep saccades during reading in adults and children. *Vision Research, 155*, 35–43. https://doi.org/10.1016/j.visres.2018.12.007

Parker, A. J., Kirkby, J. A., & Slattery, T. J. (2020). Undersweep fixations during reading in adults and children. *Journal of Experimental Child Psychology, 192*, 104788. https://doi.org/10.1016/j.jecp.2019.104788

Pedregosa, F., Varoquaux, G., Gramfort, A., Michel, V., Thirion, B., Grisel, O., Blondel, M., Prettenhofer, P., Weiss, R., Dubourg, V., et al., 2011. Scikit-learn: Machine learning in python. the Journal of machine Learning research 12, 2825–2830. https://doi:10.3389/fninf.2014.00014.

Pollatsek, A., Juhasz, B. J., Reichle, E. D., Machacek, D., & Rayner, K. (2008). Immediate and Delayed Effects of Word Frequency and Word Length on Eye Movements in Reading: A Reversed Delayed Effect of Word Length. *Journal of Experimental Psychology: Human Perception and Performance, 34*(3), 726–750. https://doi.org/10.1037/0096-1523.34.3.726

Pylyshyn, Z. W., & Storm, R. W. (1988). Tracking multiple independent targets: Evidence for a parallel tracking mechanism*. *Spatial Vision, 3*(3), 179–197. https://doi.org/10.1163/156856888x00122

Radach, D., R. ,. &. Heller. (1993). Zeilenrücksprünge und Korrektursakkaden beim Lesen von Texten (Return sweeps and corrective saccades in reading). 36th Conference of Experimentally Working Psychologists.

Radach, R., & McConkie, G. W. (1998). Chapter 4—Determinants of Fixation Positions in Words During Reading. In ["Geoffrey Underwood"] (Ed.), *Eye Guidance in Reading and Scene Perception* (pp. 77–100). Elsevier Science Ltd. https://doi.org/10.1016/b978-008043361-5/50005-5

Rawson, K. A., & Miyake, A. (2002). Does relocating information in text depend on verbal or visuospatial abilities? An individual-differences analysis. *Psychonomic Bulletin & Review, 9*(4), 801–806. https://doi.org/10.3758/bf03196338

Rayner, K. (1998). Eye Movements in Reading and Information Processing: 20 Years of Research. *Psychological Bulletin, 124*(3), 372–422. https://doi.org/10.1037/0033-2909.124.3.372

Rayner, K., & Frazier, L. (1987). Parsing temporarily ambiguous complements. *The Quarterly Journal of Experimental Psychology Section A, 39*(4), 657–673. https://doi.org/10.1080/14640748708401808

Rayner, K., Juhasz, B., Ashby, J., & Clifton, C. (2003). Inhibition of saccade return in reading. *Vision Research, 43*(9), 1027–1034. https://doi.org/10.1016/s0042-6989(03)00076-2

Slattery, T. J., & Parker, A. J. (2019). Return sweeps in reading: Processing implications of undersweep-fixations. *Psychonomic Bulletin & Review, 26*(6), 1948–1957. https://doi.org/10.3758/s13423-019-01636-3

Van der Maaten, L., Hinton, G., 2008. Visualizing data using t-sne. *Journal of machine learning research* 9 (86), 2579-2606. [link]

Vitu, G. W., F. M. ;. McConkie. (2000). Regressive saccades and word perception in adult perception. In J. Kennedy A. ,. Radach, R. ,. Pynte (Ed.), *Reading as a perceptual process* (pp. 89–118). Elsevier.

Vitu, F., McConkie, G. W., Kerr, P., & O'Regan, J. K. (2001). Fixation location effects on fixation durations during reading: An inverted optimal viewing position effect. *Vision Research, 41*(25–26), 3513–3533. https://doi.org/10.1016/s0042-6989(01)00166-3

Weger, U. W., & Inhoff, A. W. (2005). Attention and Eye Movements in Reading. *Psychological Science, 17*(3), 187–191. https://doi.org/10.1111/j.1467-9280.2006.01683.x

Werner, S., & Diedrichsen, J. (2002). The time course of spatial memory distortions. *Memory & Cognition, 30*(5), 718–730. https://doi.org/10.3758/bf03196428

Yantis, S., & Johnson, D. N. (1990). Mechanisms of Attentional Priority. *Journal of Experimental Psychology: Human Perception and Performance, 16*(4), 812–825. https://doi.org/10.1037/0096-1523.16.4.812

Yantis, S., & Jones, E. (1991). Mechanisms of attentional selection: Temporally modulated priority tags. *Perception & Psychophysics, 50*(2), 166–178. https://doi.org/10.3758/bf03212217

Yantis, S., & Jonides, J. (1990). Abrupt Visual Onsets and Selective Attention: Voluntary Versus Automatic Allocation. *Journal of Experimental Psychology: Human Perception and Performance, 16*(1), 121–134. https://doi.org/10.1037/0096-1523.16.1.121

Zechmeister, E. B., McKillip, J., Pasko, S., & Bespalec, D. (1975). Visual Memory for Place on the Page. *The Journal of General Psychology, 92*(1), 43–52. https://doi.org/10.1080/00221309.1975.9711326
