# Supplementary material for: Visuospatial, oculomotor, and executive reading skills evolve in elementary school, and errors are significant: a topological RAN study
Source: Front Psychol. 2024 Jun 6;15:1383969. doi: 10.3389/fpsyg.2024.1383969 (PMC11188999; doi:10.3389/fpsyg.2024.1383969)
Supplement: Supplementary file 2 [file Table_2.pdf]

Test	Task	Distractor	Distractor Arrangement	Item n and series	Introduction Period	Complexity Determinant	Facilitating Factors	Max Distance items	min Distance items
#1 Uncrowded Rows	Pseudo-Reading	—	—	80 - ABCD	1B	—	PPB	line spacing 132 px	3 character spaces
#2 Crowded Rows	Pseudo-Reading	Horizontal Crowding	Reduced Inter-Target distance	80 - CDAB	1B	Horizontal Crowding	PPB	Line spacing 132 px	0 character spaces - 58 approximation
#3 Reduced Antigrouping	Pseudo-Reading	Vertical Antigrouping canceled by horizontal stripes	—	80 - CDAB	1B	Target by target H Oculomotor Computation	Alternating Gray & White Horizontal Stripes<br>PPB	8 character spaces	Line spacing 14 px
#4 Vertical Antigrouping	Pseudo-Reading	Vertical Antigrouping	Grouping by serpentine columns	80 - CDAB	1B	#3 + CR computation + handling of horizontal saccades vertical error	Vertical Subitizing<br>Verbal WM<br>PPB	8 character spaces	Line spacing 12 px
#5 Colored Vertical Antigrouping	Pseudo-Reading	Vertical Antigrouping reinforced with areas and colors	Grouping by serpentine columns	80 - CDAB	1B	#4 + Grouping salience by area and color	Vertical Subitizing<br>Verbal WM<br>PPB	8 character spaces	Line spacing 12 px
#5b Dual Antigrouping	Psudo-Reading	#4 + Oblique Antigrouping	#4 + Grouping by oblique stripes	80 - CDAB	2B	#4 reinforced handling of horizontal saccades vertical error	Vertical Subitizing<br>Verbal WM<br>PPB	8 character spaces	Line spacing 12 px
#6 Vertically Crowded Columns	Vertical Reading	Vertical Crowding	Reduced inter-target Distance	80 - BADC	1B	Vertical Crowding	PPB	Inter-column > 16 character spaces	Line spacing 11 px
#7a Uncrowded Voluntary Saccades	Reading by jumping between alternate columns	—	—	20 - A	1B	—	PPB	Inter-column space > 7 character spaces	Line spacing 14
#7b Color Uncrowded Voluntary Saccades	Reading by jumping between alternate columns	Two-dimensional Attenuated Crowding	columns of black digits surround the red target-columns (3rd and 3rd-last columns)	20 - B	1B	Two-dimensional crowding reduced by target-column salience	PPB<br>Red coloring of target columns	Line spacing 14 px	Inter-column 1 character space -97 Approximation
#8a Crowded Voluntary Saccades	Reading by jumping between alternate columns	Two-Dimensional Crowding	Columns of black digits surround the 3rd and 3rd-last black target-columns	20 - C	1B	Two-Dimensional Crowding	Bidimensional Subitizing<br>Verbal WM	Line spacing 14 px	Inter-column 1 character space -97 Approximation
#8b Crowded Voluntary Saccades	Reading by jumping between alternate	Two-Dimensional Crowding	Columns of black digits surround the 3rd and 3rd-last black target-columns	20 - D	1B	Two-Dimensional Crowding	Bidimensional Subitizing<br>Verbal WM	Line spacing 14 px	Inter-column 1 character space -97 Approximation

Test	Task	Distractor	Distractor Arrangement	Item n and series	Introduction Period	Complexity Determinant	Facilitating Factors	Max Distance items	min Distance items
	columns								
#9 Switch online	Serpentine Path	—	—	88 - AB'CD	1B	Target by target Oculomotor / span flexibility	PPB	4 character spaces	Line spacing 12 px
#10 Switch in Crowd	Serpentine Path	Two-Dimensional Crowding	Pseudo-Digits surrounding the serpentine path	88 - AB'CD	1B	#9 + two-dimensional crowding	PPB	4 character spaces	Line spacing 12 px
#11 Reverse Reading	Reverse Reading (derived from #3)	Vertical antigrouping canceled by horizontal stripes	—	80 - CDAB	2B	Target by target Reverse Oculomotor computation	Vertical Subitizing<br>Verbal WM<br>PPB	8 character spaces	Line spacing 14 px
#12 Antigrouping Reverse Reading	Reverse Reading (derived from #4)	Vertical Antigrouping	Grouping by serpentine columns	80 - CDAB	2B	#11 + Reverse CR + handling of horizontal saccades vertical error	Vertical Subitizing<br>Verbal WM<br>PPB	8 character spaces	Line spacing 12 px
#13 Colored Antigrouping Reverse Reading	Reverse Reading (derived from #5)	Vertical AntiGrouping reinforced with areas and colors	Grouping by serpentine columns	80 - CDAB	2B	#12 + Grouping salience by area and color	Vertical Subitizing<br>Verbal WM<br>PPB	8 character spaces	Line spacing 12 px

Table 3. Subtest Description. [legend: CR = Carriage Return; PPB = Parafoveal Preview Benefit; WM = Working Memory].

AltText

Table 3 presents a detailed description of the various subtests. It includes columns that list the number and name of each subtest, the specific task required by the subtest (e.g., Pseudo-Reading) if contemplated, the type of distractor (e.g., horizontal crowding), and the arrangement of distractors (e.g., reduced inter-target distance), the number of items and the pseudo-randomized number series used (e.g., 80 - ABCD), the school period in which the test was introduced (e.g., 1B which would indicate 1st-grade 3rd quarter), the determinant of the visuospatial complexity of the subtest (e.g., horizontal crowding), the factors facilitating the performance of the subtest (e.g., PPB = Parafoveal Preview Benefit); the maximum distance between digits on the subtest (e.g., line spacing 132 px) and the minimum distance between digits (e.g., three character spaces). Key abbreviations such as CR for Carriage Return, PPB for Parafoveal Preview Benefit, and WM for Working Memory are elucidated. This table is instrumental in understanding the specific assessments and metrics used in the study.
